# A Biophysical Model for Analysis of Transcription Factor Interaction and Binding Site Arrangement from Genome-Wide Binding Data

**DOI:** 10.1371/journal.pone.0008155

**Published:** 2009-12-01

**Authors:** Xin He, Chieh-Chun Chen, Feng Hong, Fang Fang, Saurabh Sinha, Huck-Hui Ng, Sheng Zhong

**Affiliations:** 1 Department of Computer Science, University of Illinois at Urbana-Champaign, Champaign, Illinois, United States of America; 2 Department of Bioengineering, University of Illinois at Urbana-Champaign, Champaign, Illinois, United States of America; 3 Department of Statistics, University of Illinois at Urbana-Champaign, Champaign, Illinois, United States of America; 4 Gene Regulation Laboratory, Genome Institute of Singapore, Singapore, Singapore; National University of Ireland Galway, Ireland

## Abstract

**Background:**

How transcription factors (TFs) interact with *cis*-regulatory sequences and interact with each other is a fundamental, but not well understood, aspect of gene regulation.

**Methodology/Principal Findings:**

We present a computational method to address this question, relying on the established biophysical principles. This method, STAP (sequence to affinity prediction), takes into account all combinations and configurations of strong and weak binding sites to analyze large scale transcription factor (TF)-DNA binding data to discover cooperative interactions among TFs, infer sequence rules of interaction and predict TF target genes in new conditions with no TF-DNA binding data. The distinctions between STAP and other statistical approaches for analyzing *cis*-regulatory sequences include the utility of physical principles and the treatment of the DNA binding data as quantitative representation of binding strengths. Applying this method to the ChIP-seq data of 12 TFs in mouse embryonic stem (ES) cells, we found that the strength of TF-DNA binding could be significantly modulated by cooperative interactions among TFs with adjacent binding sites. However, further analysis on five putatively interacting TF pairs suggests that such interactions may be relatively insensitive to the distance and orientation of binding sites. Testing a set of putative Nanog motifs, STAP showed that a novel Nanog motif could better explain the ChIP-seq data than previously published ones. We then experimentally tested and verified the new Nanog motif. A series of comparisons showed that STAP has more predictive power than several state-of-the-art methods for *cis*-regulatory sequence analysis. We took advantage of this power to study the evolution of TF-target relationship in Drosophila. By learning the TF-DNA interaction models from the ChIP-chip data of *D. melanogaster* (Mel) and applying them to the genome of D. *pseudoobscura* (Pse), we found that only about half of the sequences strongly bound by TFs in Mel have high binding affinities in Pse. We show that prediction of functional TF targets from ChIP-chip data can be improved by using the conservation of STAP predicted affinities as an additional filter.

**Conclusions/Significance:**

STAP is an effective method to analyze binding site arrangements, TF cooperativity, and TF target genes from genome-wide TF-DNA binding data.

## Introduction

The spatial-temporal patterns of gene expression are controlled by *cis*-regulatory sequences [1], through binding of transcription factors (TFs) to specific sites in these sequences. Numerous studies point out that the final transcriptional “read-out” is determined, not by an individual TF, but by the combinatorial interactions of multiple TFs with DNA. Most notably, in developmental genes, multiple binding sites of different TFs are often located close to each other in genomes, forming so called *cis*-regulatory modules (CRMs), and work together to generate precise expression patterns [2].

Sequence-specific binding of TF molecules to DNA has been well studied, both in theory [3] and in practice [4]. In contrast, the interactions between TF molecules that enhance or inhibit their DNA binding affinities or transcriptional effects are not well understood. Although the importance of cooperative interactions among TF molecules in gene regulation were clearly demonstrated [5]–[8], it is not clear, at a quantitative level, what are the roles of such interactions, and in most systems the identities of interacting TFs remain unknown. In cases where multiple TF molecules do interact, it is generally unknown how the spatial organization of their binding sites affects DNA binding. Some studies suggest that binding sites must be arranged in specific ways, following “grammar-like rules” [9], [10] in order for them to interact properly; others provide evidence of a flexible organization of regulatory sequences [11], [12]. Knowledge of the role of TF interactions and how they interact will be central to our understanding of gene regulation.

Genome-wide DNA-binding data from chromatin immunoprecipitation followed by either genome tiling array analysis (ChIP-chip) or sequencing (ChIP-seq), provide an opportunity to address the above-mentioned problems quantitatively [13], [14]. DNA-binding by TFs is a key step in transcriptional regulation, thus modeling combinatorial TF-DNA interactions will serve as a bridge to understanding the complex transcriptional process. Focusing on ChIP-based data, instead of gene expression data, simplifies the task at hand. Gene expression is often accomplished through an intricate process involving not only TF-DNA interactions, but also chromatin remodeling, epigenetic modifications, communications among multiple enhancers, etc [15]. For this reason, several studies have argued for studying combinatorial interactions among TFs using ChIP-based technologies [16], [17].

The central task of this work is to build a predictive model of TF binding affinity from DNA sequences, incorporating both TF-DNA and TF-TF interactions. This would allow us to learn how cooperative interactions among TFs may contribute to their DNA binding affinities. By varying the assumptions of TF interactions and observing their effects on the model predictability, one may be able to understand the details of how binding site arrangements affect interactions. Moreover, a model trained from one set of sequences in one situation can be applied to a different setting to make more predictions about TF targets. This extrapolative ability will be useful, for instance, when we only have TF binding data for part of the genome (e.g. only promoters) and want to identify more TF targets (a large portion of regulatory sequences may lie outside the promoter regions in higher organisms). In one of the analyses, we applied the binding models learned from one genome to predict affinities of the orthologous sequences in a related organism. Such predictions facilitate the analysis of the evolution of TF binding even when ChIP-chip or ChIP-seq data are available in only one organism.

A number of computational methods have been proposed to study the TF binding profiles [18], [19] and combinatorial aspect of gene regulation through predictive models [20]. Typically, these methods attempt to extract information from statistical patterns in DNA sequences, e.g., the occurrence of sequence motifs. Various techniques from statistical learning, such as Bayesian networks [10], multivariate regression [19], [21], [22], decision trees [20], regression trees [23], SVM and artificial neural networks [24], were applied to extract important features from sequences, using either gene expression or ChIP-chip data. However, these methods do not reflect underlying physical principles. As such, it is not clear to what extent their assumptions, e.g., additivity of different features, are valid. Additionally, important sequence features, such as interactions among adjacent binding sites, are often not represented in these approaches. Quantitative methods that are not based on predictive modeling are also available for analyzing ChIP-chip or ChIP-seq data for the purpose of identifying binding sites in the data [25], [26] or patterns of co-occurrence of motifs [27], [28]. These methods serve somewhat different goals and do not offer the benefits of predictive models. Interested readers are referred to recent reviews [14], [20].

By directly modeling the underlying processes, a biophysics-based approach can overcome many limitations of the statistical methods mentioned above. Shea and Ackers [29] and Buchler et al. [30] pioneered the use of thermodynamic principles in the study of regulatory mechanisms. A number of recent studies applied these principles to model expression data on promoters/enhancers [6], [23], [31]–[33] or TF-DNA binding data from ChIP-chip experiments [18], [19], [34]. However, these methods have not adequately addressed the interaction of multiple transcription factors with each other and with DNA. Also, most of these studies focused on individual regulatory sequences [31]–[33] rather than genome-wide data, while others have taken the route of simulations [33], or studied artificial promoters [6], which are by design far simpler than natural systems. In summary, no existing work has provided a quantitative framework to analyze genome-wide TF-DNA binding data based on realistic biophysical modeling, especially of combinatorial interaction among multiple TFs and their DNA binding sites.

We developed a novel method, called STAP (Sequence To Affinity Prediction), to analyze large scale TF-DNA binding data. The heart of this method is a thermodynamic model adapted from earlier theoretical studies [29], [30]. The key novel feature of STAP is the explicit treatment of cooperative interactions among different TF molecules. Different from existing thermodynamic models, STAP explicitly expresses the expected number of TFs bound to a regulatory sequence, and thus it is directly applicable to analyze binding intensities reflected in whole-genome binding data. In addition, our specially developed computational techniques based on dynamic programming will enable the model to be efficiently applied to complex sequences and large scale data. Another main feature of STAP is the utility of genome-wide binding data not only as binary indicators of TF binding regions, as been done by most existing studies, but also as quantitative measurements of the binding strengths. Thus, more information from these data will be utilized by this new method. STAP was applied to analyze the ChIP-seq data of 12 TFs in mouse embryonic stem cells (ESCs) [35] and the ChIP-chip data of two TFs involved in fruit fly blastoderm development [16]. The analysis results demonstrated the effectiveness of the new method to address issues in combinatorial gene regulation using genome-wide binding data.

## Results

### ChIP-Seq Data Can Be Quantitatively Reproduced

We hypothesized that ChIP-seq data quantitatively reflect the binding strength between the TF and the respective genomic binding regions, and therefore should be quantitatively reproducible. To verify this hypothesis, we randomly picked 28 Nanog ChIP-seq detected binding regions from [35] and repeated the ChIP experiments in E14 mouse ES cells. We used real-time qPCR to quantify the ChIP precipitated DNA on the 28 pre-selected regions. The ChIP-seq and ChIP-qPCR signals exhibited a strong correlation (*r^2^* = 0.656, Figure S1). We performed the same experiment on 11 SUZ12 binding regions from ChIP-seq data and similarly found a strong correlation (*r^2^* = 0.792, Figure S1). These data suggest that the counts of overlapping ChIP-seq tags are quantitatively reproducible by independent experiments. Thus it becomes possible to model and utilize the quantitative nature of ChIP-seq data for investigating the biophysical rules of protein-protein and protein-DNA interaction.

### Transcription Factors Are Extensively Co-Localized

We studied ChIP-seq data on 12 TFs active in embryonic stems cells [35]: cMyc, CTCF, E2f1, Esrrb, Klf4, Nanog, nMyc, Oct4, Sox2, STAT3, Tcfcp2l1 and Zfx. Combinatorial gene regulation leads to a statistical tendency of multiple factors to bind to proximally located sites, a phenomenon we call TF “co-localization”. We developed a statistical test for co-localization of TF pairs (Text S1) and found extensive evidence for this phenomenon (Table S1). The majority (121) of all 132 possible pairs show significant co-localization (*p*<0.01, Pearson's χ^2^ test). Our results are broadly consistent with those of Chen et al. [35], which also revealed extensive co-localization (though no statistical tests were provided). In summary, both analyses strongly suggest a combinatorial mode of action by multiple factors.

### A Biophysical Model of TF Binding to DNA Sequences

**Figure 1:**
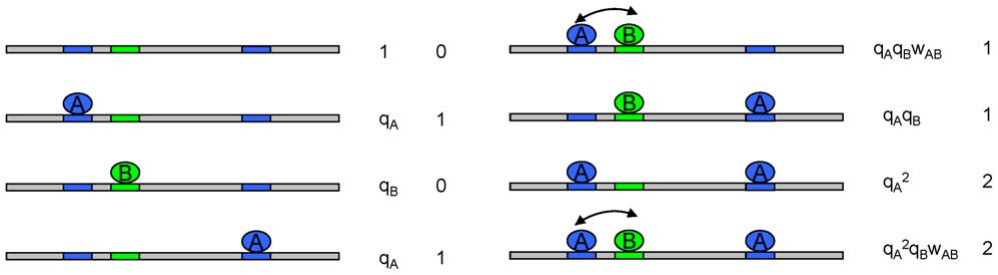
Model of cooperative DNA binding. The sequence contains three binding sites, two for factor *A*, and one for factor *B*. All eight configurations of this sequence, in terms of binding site occupancy, are shown. The arrow connecting two adjacent bound molecules indicates cooperative interaction. For each configuration, the first column represents the weight, i.e., un-normalized probability, and the second column represents the number of bound molecules of *A*. The parameters in the weight terms are: q_A_ (q_B_) – strength of factor *A* (*B*) binding to DNA; w_AB_ – strength of the interaction between *A* and *B*. The binding affinity of *A* to this sequence is the average of the second column, weighted by the first column.

A possible explanation for TF co-localization is that DNA-binding of one factor helps recruit another factor to its binding site, through favorable TF-TF interaction. (Note that the binding sites in this paper refer to 10–20 bp regions actually occupied by TFs, while other papers may refer to putatively larger regions identified in ChIP-chip or ChIP-seq experiments – these will be called TF-bound regions in our paper). Thus, when co-localized, both factors may access the DNA with higher affinity than their individual binding sites alone would allow. We adapted the biophysical model from [30] that incorporates such cooperative binding, for the purpose of analyzing TF-DNA binding data. Given a transcription factor (called “TF_exp_”), our goal is to predict the binding affinity of TF_exp_ to any sequence. The basic assumption is that many putative binding sites, including the sites of TF_exp_ and of other factors, not just the single best match, may contribute to interaction of this sequence to TF_exp_. Indeed, the importance of weak binding sites and cooperative interactions has been supported by a number of recent studies [6], [18], [23], [34]. Under this picture: binding sites of TF_exp_ directly attract TF_exp_, and sites of other factors may interact cooperatively with TF_exp_, thus indirectly recruiting TF_exp_. The cooperative interactions may occur among adjacent binding sites of the same TF (self-cooperativity) or of different TFs (heterotypic cooperativity). Thermodynamically, each binding site of a sequence may be occupied or not, thus a sequence with 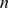 sites exists in 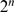 states, where each state represents the occupancy status of all sites (Figure 1). The probability of a state depends on interactions of TFs with their binding sites, as well as TF-TF interactions, as quantified by Equation (2) in Methods. Following earlier work on ChIP-chip data analysis [19], [34], we assume that the binding affinity of TF_exp_ to this sequence is proportional to the average number of TF_exp_ molecules occupying their sites, over all states weighted by their probabilities (Figure 1). Note that the number of states is exponential to the number of binding sites, thus it is computationally difficult to calculate the binding affinities of complex sequences by the brute-force method. We developed a dynamic programming algorithm to carry out the computation efficiently. The details of the model and the algorithm can be found in Methods.

When analyzing the genome-wide binding data of some TF (hereafter called the primary factor), the goal is to learn the TFs (called cooperative factors) that interact with this factor, as well as the relevant model parameters. The STAP model is fit by maximizing the Pearson's correlation coefficient between the predicted binding affinities and the overlapping ChIP-seq counts (or ChIP-chip intensities). To search for interacting factors, we iterate the motifs in a motif collection, such as the JASPAR database [36]. Each motif in this collection is tested by whether adding this motif to the STAP model with only the primary factor will significantly improve the Pearson's correlation coefficient. The significance of this improvement is assessed by using a large number of randomized motifs as negative controls. After all cooperative factors are learned, and STAP re-trains the model parameters. The STAP model is designed for analyzing ChIP data from a single TF; a variation of STAP is developed for simultaneously analyze ChIP data from several TFs (see “Exploring the effects of binding sites arrangement”).

### ChIP-Seq Data Reveals a Novel Characterization of Nanog Binding Specificity

**Figure 2:**
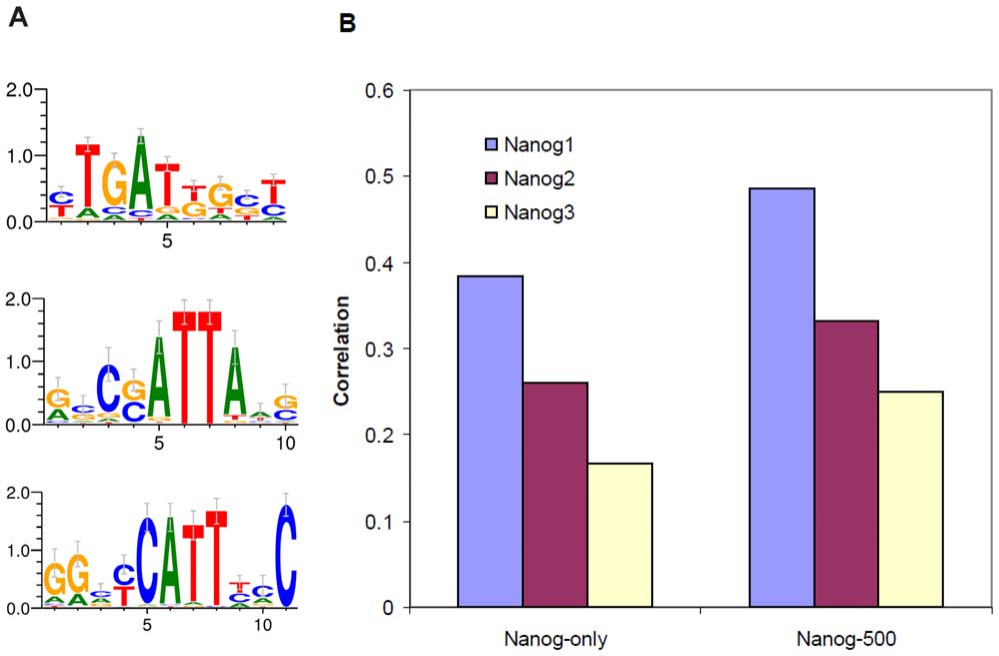
Comparison of three versions of the Nanog motif. (A) Nanog1 – the motif learned from the sequences bound by Nanog, but not Oct4 and Sox2, in the ChIP-seq data; Nanog2 – the motif in [40]; Nanog3 – the motif in [41]. (B) Performance of models using three different versions of the motif, measured by the correlation between model predictions and observations. The models are applied to two different sets of data. Nanog-only: the sequences bound by Nanog, but not Oct4 and Sox2; Nanog-500: the 500 sequences with strongest binding to Nanog.

Our method needs to use motifs of TFs, representing binding specificities, to identify putative binding sites in target sequences (though it is theoretically possible to learn novel motifs under our framework, similar to [19]). So at the first step, we identified the motifs of the 12 TFs. For each factor, we ran the MEME program [37] on the top 100 regions (ranked by tag counts) detected in the ChIP-seq experiments. These motifs (Figure S2) are by and large similar to those reported in the original ChIP-seq paper [35]. However, we noted that the motifs of Oct4, Sox2 and Nanog, learned by [35] were remarkably similar to each other. We hypothesized that this similarity was due to co-localization of the factors, which resulted in similar collections of genomic regions being used for enrichment-based motif finding. To test this hypotheses, we used sequences bound exclusively by each of these three factors and performed MEME analysis again (NestedMICA [38] and Gibbs sampler [39] gave similar results). The resulting Oct4 and Sox2 motifs are similar to the corresponding parts of the previously identified Oct4-Sox2 joint motif, while the Nanog motif is different (Figure 2A, Nanog1). We also note that several other DNA binding profiles of Nanog were reported from previous studies [35], [40], [41], but they do not resemble each other. Inspired by the importance of Nanog as an essential regulator in ESC proliferation and self-renewal [40], we set out to characterize the binding specificity of Nanog using a combination of computational and experimental approaches.

Even though STAP was not designed for *de novo* motif finding, it is applicable to compare multiple motifs of the same factor. By setting these motifs as alternative inputs and comparing the model fit to genome-wide binding data, the best motif can be recognized. We applied this strategy to the new Nanog motif as well as two previously published ones (Nanog2 [40] and Nanog3 [41], Figure 2A) to test if the new motif better explains the ChIP-seq data. The new Nanog motif resulted in a higher correlation than the other two in the sequences bound only by Nanog, but not Oct4 and Sox2 (Figure 2B, Nanog-only), providing initial support to the novel Nanog motif. In a second test, we utilized STAP's capability of analyzing cases where multiple factors are bound. As discussed before, the enrichment of Oct4 and Sox2 binding sites in the Nanog-bound sequences tend to confuse the motif discovery tools. This obstacle was resolved by setting Oct4 and Sox2 as cooperative factors, and varying the candidate primary motif. In this way, the difference of results was attributed to the different Nanog motifs, with the effects of Oct4 and Sox2 sites automatically disentangled. Again, the new Nanog motif provided a significantly better fit to the ChIP-seq counts of the Nanog bound sequences than the other motifs (Figure 2B, Nanog-500). In addition, the fitting of observations with the new Nanog motif is highly significant under a test using randomized motifs (Figures S3).

The enhanced model fitting with the new Nanog motif tempted us to experimentally test it. Electrophoretic mobility shift assay (EMSA) was used to test the novel Nanog motif (Text S1). First, from the Nanog ChIP-seq positive regions, we randomly selected five sequences that match the new Nanog motif but do not match the Oct4-Sox2 joint motif (Table S2). EMSA produced the same band from these five sequences, which also match the band produced from a positive control region known to interact with Nanog (Figure S4). Second, we performed a series of point mutations to a wild type sequence that matches the new Nanog motif (Table S3). Since “TGA” from position 2 to position 5 is the most conserved part of the new motif, we focused the point mutations to these three positions. Mutating the “TGA” core of the motif completely abolished the binding. Except the “G to A” mutation on position 3, the other six point mutations to the “TGA” core severely reduced or completely abolished binding (Table S3). These mutation results were not affected by the wild-type Nanog binding site (Figure S5). We also compared binding specificities of the DNA binding domain of Nanog and the whole Nanog protein. No difference was found in all EMSA experiments. In summary, the EMSA data on the five wild-type sequences and point mutations were consistent with the notion that Nanog binds to the novel motif.

### Cooperativity among TFs Is Frequently Associated with DNA Binding

We next identified cooperative interactions among TFs for DNA binding. For each ChIP-seq experiment, we created training and testing data sets, each consisting of 500 bound and 500 randomly chosen unbound sequences. STAP was applied to learn the significant cooperative factors (among all eleven possible candidates) for each experiment in the training data, following the procedure described in Methods (Table 1). This analysis reproduced some known (functional or physical) interactions, including Sox2-Oct4 [42] and cMyc-E2f1 [43]. In addition, the pairs Nanog-Esrrb and Oct4-Esrrb, which were reported to interact in ESCs [44], [45], exhibited small *p* values (0.06 and 0.08 respectively). The results also suggested that Klf4 may cooperate with a number of other factors, i.e., Oct4, Sox2, Nanog and STAT3. Klf4 facilitates self-renewal of ESCs and promotes the efficiency of inducing pluripotency [46], through mechanisms that are not completely clear. The predicted cooperative interactions between Klf4 and other key TFs may underlie the function of Klf4. Using the testing data, we were able to confirm most of predicted interactions. All cooperative pairs, except CTCF as a co-factor of Klf4, improved the basic models where only the primary factor was used, in the testing data, suggesting that the results were not due to model overfitting (Table S4). These results seem to suggest that even though eleven motifs were tested simultaneously at each experiment, the significance threshold (*p* value = 0.05) is stringent in practice. We therefore chose not to further correct for multiple hypothesis testing.

**Table 1:** Cooperative interactions among factors are important in explaining TF-DNA binding data.

Factor	Non-coop. Model	Coop. Model	Improvement	Significant Coop. Factor (p-value)
cMyc	0.57	0.82	44%	E2f1(0.004), Klf4(0.04), Zfx(0.033)
CTCF	0.75	0.81	7%	
E2f1	0.50	0.66	31%	Nanog(0.048)
Esrrb	0.62	0.78	26%	Zfx(0.003)
Klf4	0.58	0.74	28%	CTCF(0)
Nanog	0.24	0.50	107%	Sox2(0), Klf4(0.012), Zfx(0.05)
nMyc	0.67	0.83	23%	E2f1(0.005)
Oct4	0.45	0.56	22%	E2f1(0.029), Klf4(0.032), Zfx(0.017)
Sox2	0.50	0.62	24%	Klf4(0.014), Oct4(0.039), Zfx(0.045)
STAT3	0.52	0.65	24%	Klf4(0.004), E2f1(0.049), Zfx(0.039)
Tcfcp2l1	0.74	0.76	3%	Esrrb(0.121)
Zfx	0.70	0.71	1%	

In non-cooperative (non-coop.) model, only the motif of TF_exp_ is used for fitting the data and no cooperativity is allows. In cooperative (coop.) model, both the motif of TF_exp_ and the motifs of significant cooperative factors are used, and the cooperative interactions among factors, including the homotypic interaction, are allowed. The performance of a model is measured by the Pearson correlation between model predictions and observations in an independent testing data (not used for training the models). Significance of a cooperative factor is determined through comparison with a large number of randomized motifs. Only the factors with *p* value less than or equal to 0.05 are shown.

After training a single binding model for each factor using all its significant cooperative factors, we compared the effectiveness of this cooperative model with the “non-cooperative model” where no cooperative interaction (not even self-cooperativity) is allowed, in the independent testing data. For most factors, incorporating TF interactions substantially improved the predictive ability of the models (Table 1). These results were consistent with our initial intuition that incorporating TF-TF interactions may improve the predictive model, and hence we recommend the final trained model for predictive purposes (to classify a new sequence as being bound to the TF or not). Interestingly, for CTCF and to a small extent Zfx, the cooperative model outperformed the non-cooperative one, even though no significant cooperative factor was found, suggesting that self-cooperativity may play a role in these factors.

To explore other interacting factors that did not have genome-wide binding data, we repeated the above analysis using motifs from the JASPAR database [36], in addition to the motifs in this dataset. We found several cooperative pairs involving factors not in the original TF list in ChIP-seq experiments, including for example, Elk1-Klf4, SP1-Nanog, Zfx-TFAP2A and GABPA-Oct4. The most interesting pair seems to be GABPA-Oct4. GABPA expression is known to be induced in undifferentiated ES cells and its expression decreases during differentiation [47]. Moreover, GABPA has been shown to regulate the expression of Oct4 in mouse ESCs [48]. Thus, it would be interesting to test experimentally how GABPA is related to the function of Oct4. This is an example where our method can be utilized to automatically discover biologically plausible hypothesis from existing resources of DNA binding and motif data.

### STAP Improves Prediction of TF Targets over Existing Methods

An intended application of STAP is to use the learned binding model to predict affinities of unseen sequences to a set of TFs. An initial support to this application came from the results above showing incorporating cooperative interactions were more predictive than simple models without interactions (Table 1). We then compared STAP with the existing methods that are also capable of predicting TF target sequences. Two popular programs were chosen for this purpose, Cluster-Buster [49] and Stubb [50]. Both programs take a set of TF motifs as input, and predict if some binding site clusters appear in a test sequence. To use these programs to predict the targets of some TF, it was necessary to obtain the relevant motifs (in addition to the motif of this TF). Neither program provides such capabilities, and therefore we used another program Clover for this purpose [51]. In summary, the executed procedure of applying these two programs was: first learn all overrepresented motifs using Clover from TF-bound sequences in the training data, and then classify all sequences in the test data using Cluster-Buster or Stubb (the same training and testing data as used in the previous section). We evaluated the classification performance with the standard ROC curves, which quantifies the tradeoff of specificity and sensitivity as the classification threshold varies.

**Figure 3:**
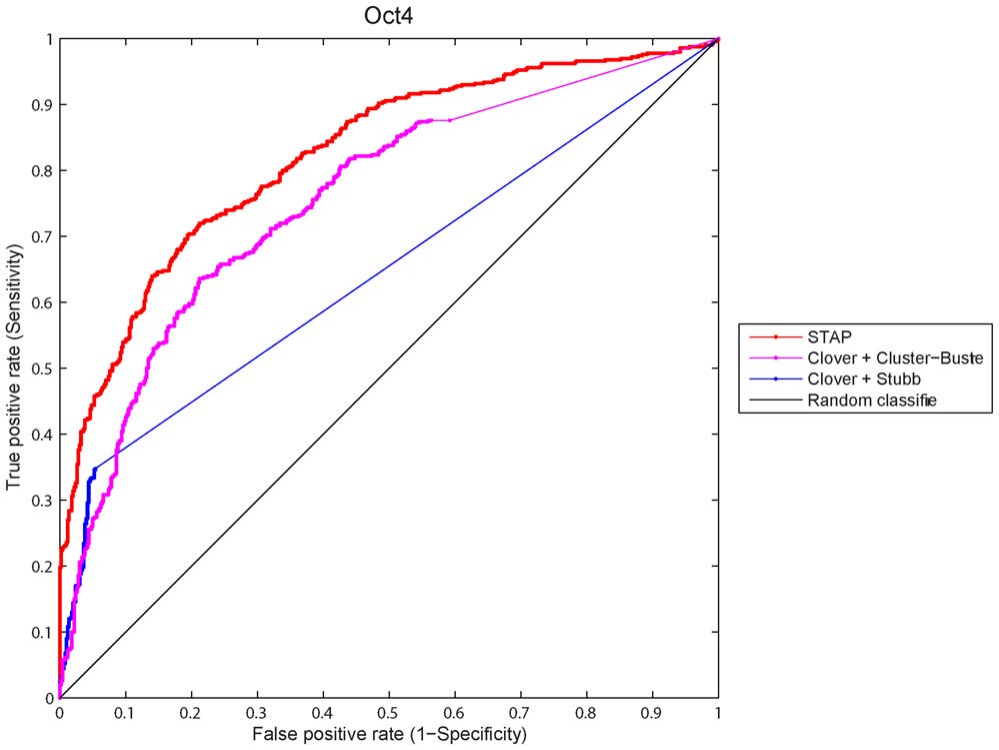
ROC curves comparing the performance of three methods for classification of Oct4 target sequences in the ChIP-seq data of Oct4. For evaluation of Cluster-Buster and Stubb, the Clover program is run first on the training data to extract a set of overrepresented motifs, which will be used as inputs of Cluster-Buster and Stubb.

Clover identified a number of overrepresented motifs from the collection of 12 motifs of the 12 assayed TFs (Table S5). These results were similar to STAP's predictions in some aspects: both predicted few interacting factors for CTCF, E2f1 and Esrrb, and some pairs were predicted by both including Nanog-Sox2 and Tcfcp2l1-Esrrb. But Clover and STAP generated quite different results on other factors (compare Table 1 and Table S5). We noticed that Clover results were largely parallel to the co-localization results in [35], with Oct4, Sox2, Nanog and Esrrb forming a cluster of mutually interacting factors. Clover effectively identified motifs whose presence in the training sequences could not be explained by chance alone, regardless of whether these motifs actually facilitate binding of the primary factor. We comment on these different ways of defining “interacting” factors in Discussion. For now, this motif set was simply applied to predict TF targets by Cluster-Buster and Stubb. In almost all cases, STAP better classified the sequences in the testing data than the other two programs (see Figure 3 for the Oct4 result, and Figure S6 for the rest).

### Exploring the Effect of Binding Site Arrangement

How binding sites are arranged in a regulatory sequence is an important, but poorly understood aspect of combinatorial gene regulation. Our biophysical model includes a component that describes how the strength of interaction between bound TF molecules depends on the arrangement (distance and relative orientation) of their respective binding sites. By varying this component, we tested if the data supports a particular mode of TF interaction over others. In each of the three models we studied (see Methods), we assume a maximum distance *d*_max_ between the two bound factors, beyond which there is no interaction. Under the “Binary” model, which is also our default model used in the analysis reported above, the strength of interaction is constant within the range of 0 to *d*_ma*x*_. Under the “Linear” model, the interaction is stronger when the two cooperative sites are closer. For both Binary and Linear models, there may be an orientation bias: the interaction of two factors may depend on the relative orientation of the two binding sites. The extent to which one orientation is favored is encoded by a bias parameter. Finally, under the “Periodic” model, the strength of interaction is a periodic function of the distance. This periodicity has been reported in a few cases before and often corresponds to the helical period of DNA molecules [52], [53].

Because the analysis here is focused on likely subtle details of binding site arrangements, we decided to work on the TF pairs with the strongest evidence of cooperative interactions. From Table S4, we chose the most significant cooperative factor, as defined by *p* values, for each primary TF (removing those not showing large improvements in the testing data). Further combining these significant pairs with prior knowledge of interacting TFs in ESCs led to five TF pairs: cMyc-E2F1, Nanog-Esrrb, Oct4-Zfx, Sox2-Oct4 and STAT3-Klf4. The overall patterns from the five pairs were very similar. Shown here are the results of Sox2-Oct4 and Nanog-Esrrb, both interactions suggested before by experimental work [44], [54], and the rest are presented in Figure S7. We note that the model fitting procedure is different from the other parts of the paper. Instead of learning the model separately for each TF, we learn a single model, where the same interaction parameters are used for the data of both factors (see Methods). This procedure was designed to maximize the use of data and enhance the signals.

**Figure 4:**
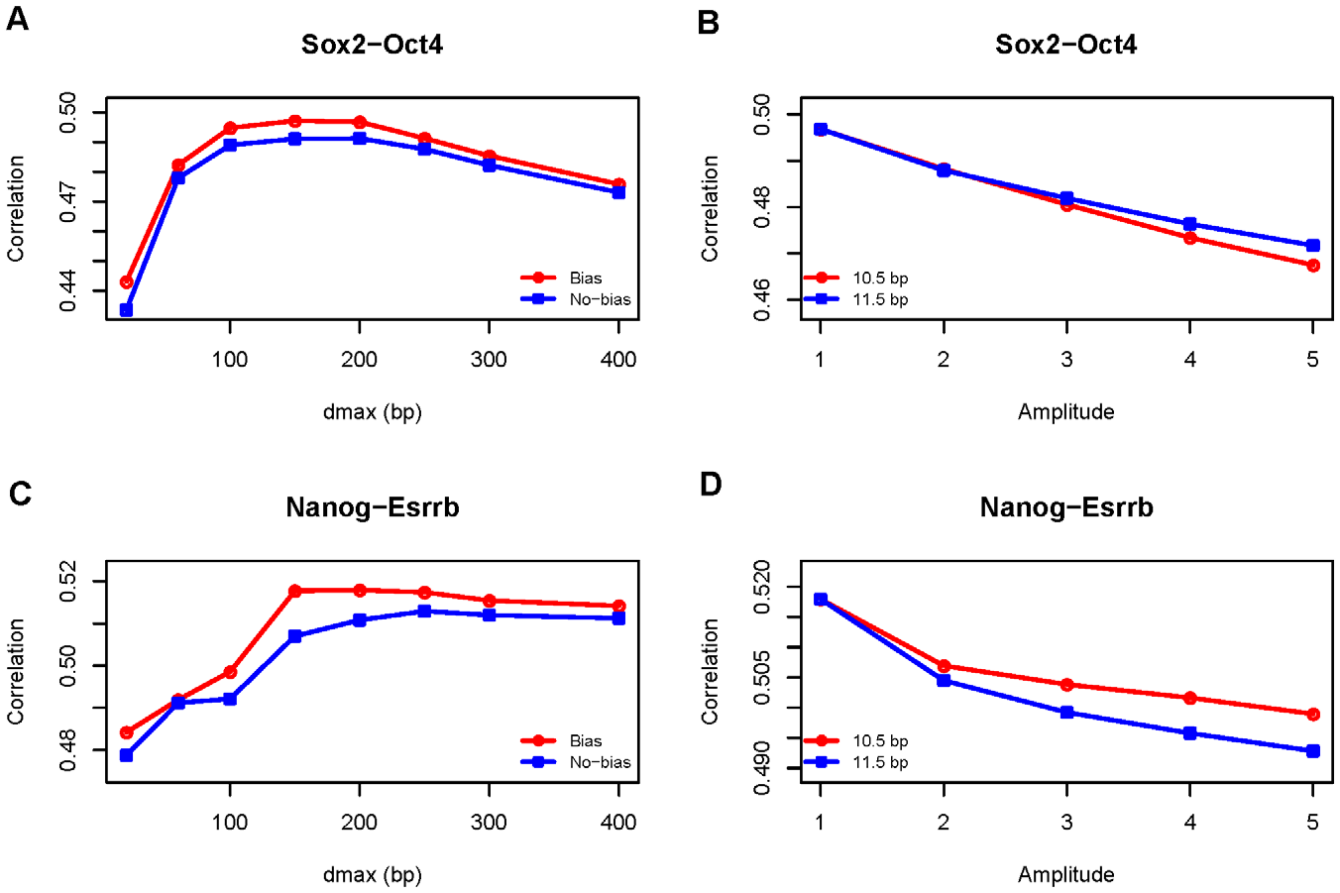
The effect of binding site arrangement on TF interactions. (A,C) Under the Binary model of interaction, the relationship between model performances, measured by correlation between predictions and observations, and the distance parameter (maximum distance, measured in bp, where two factors can interact along DNA sequence). For each value of the distance parameter, two models are compared: one in which the orientation bias parameter is optimized, and the other not allowing the bias. (B,D) Under the Periodic model of interaction, the relationship between model performances and the amplitude parameter (the change of the interaction strength within a period). Only two values of periodicity are shown.

The first studied was the Binary model of cooperative interaction. We varied the *d*_max_ parameter and for each value of *d*_max_, we optimized the orientation bias parameter and compared this optimized model with the one without bias. Small orientation bias was found in the cases of Nanog-Esrrb, Sox2-Oct4 and cMyc-E2f1, where the free energy that penalizes one orientation is about 20% of the interaction free energy, and no such bias was detected for STAT3-Klf4 and Oct4-Zfx. What is more revealing is that the performance of the models which optimized the bias parameter was close to the one without bias (Figure 4A, 4C, Figure S). The differences in terms of correlation coefficients are less than 1% in most cases (except Nanog-Esrrb, which reaches about 2%). In contrast, the parameter *d*_max_ plays a much larger role (Figure 4A, 4C, Figure S7). More tested TF interactions occur in the range of 150–200 bp. (Figure 4A, Figure S7). Next, we observed that the Linear model did not improve the predictability (the Linear model actually does better only in the case of Oct4-Zfx, but the improvement is less than 1%), suggesting that interaction between two factors does not decrease significantly with distance. Finally, for the Periodic model, we varied the periodicity from 10.0 to 12.0 bp (corresponding roughly to the range of DNA helix), and for each of these values, we also varied the amplitude parameter, which is a measure of the strength of periodicity (see Methods). Similar to the results from the Linear model, we found that this more complex model is no better than the simpler Binary model. In fact, the performance of the Periodic model always decreases when the amplitude parameter is increased under all values of periodicity we tested, suggesting that the interactions are not periodic for these pairs (Figure 4B, 4D, Figure S7). All these results: the lack of clear orientation bias, tolerance to distance change and the lack of periodicity together seem to indicate that binding site interactions do not follow strict rules, at least in these tested cases.

### Application to *Drosophila* ChIP-Chip Data of Segmentation Factors

This section presents the results on testing STAP for its capability of making cross-species extrapolations. We constructed binding models of the TFs regulating pattern formation in the early embryonic development of *D. melanogaster* (Mel) and applied them to the genome of *D. pseudoobscura* (Pse). While the original paper reported the ChIP-chip data of six TFs, we focused on two of the most well characterized, Bcd and Kr, as the other factors did not have sufficient amount of data or reliable binding profiles [31], [55]. We trained the binding models of Bcd and Kr in 1000 Mel sequences, half from bound sequences at 1% FDR level, and the other half from random unbound sequences. These models were then applied to the Pse orthologs of all bound sequences (at 25% FDR level) and 250 random unbound sequences. A sequence was considered conserved if the predicted binding affinity of its ortholog was above certain threshold (learned from the training data in Mel).

STAP successfully predicted binding affinities of orthologous sequences in the Pse genome. We assumed that the majority of the random unbound sequences should remain unbound in Pse. Since STAP predicted that 13% (for Bcd) and 22% (for Kr) of these random sequences have high binding affinities (Table 2), the specificities of STAP predictions were no smaller than 87% and 78%, respectively. Based on the observation that many known enhancers are also functional in Pse [23], [56], [57], we estimated the model sensitivities at 83% and 48%, respectively, corresponding to the fractions of known enhancers that have conserved orthologs in Pse (Table 2). We note that some enhancers do not have orthologous sequences in Pse (from UCSC alignment), thus the classification of these enhancers as having non-conserved affinity is not a fault of our prediction method. If adjusting for these cases, the model sensitivities would become 91% (Bcd) and 62% (Kr). Overall, STAP achieved medium to high sensitivities for predicting Bcd and Kr targets in the Pse genome with low false positive rates.

**Table 2:** The conservation of binding affinities to Bcd and Kr of different groups of sequences in *D. melanogaster*.

Sequences	Bcd	Kr
Random	0.13 (32/250)	0.22 (54/250)
Enhancers	0.83 (29/35)	0.48 (16/33)
Bound (1% FDR)	0.45 (310/692)	0.34 (685/2001)
Bound (1% FDR) and expressed	0.43 (141/331)	0.33 (205/621)

A sequence is conserved if the predicted affinity of its orthologous sequence in *D. pseudoobscura* is above a specified threshold. Shown in each cell is the fraction of conserved sequences, and in parenthesis, the number of conserved sequences and the total number of sequences in that group. Random: random unbound sequences; Enhancers: known blastoderm enhancers that overlap with some bound regions (25% FDR); Bound: all bound sequences at 1% FDR; Bound and expressed: all bound sequences that are also adjacent to some genes transcribed in blastoderm.

Interestingly, STAP predicted that the binding affinities of a large fraction of TF-bound sequences are not conserved. Among all bound regions at 1% FDR level, only 45% (Bcd) and 34% (Kr) were predicted to have conserved affinities in Pse (Table 2), and the fraction of conservation for bound sequences at 25% FDR was even lower. Such a low level of conservation could be attributed to errors in model prediction, where some conserved sequences might be missed by STAP predictions. However, this alone cannot account for the low conservation level we observed, as the numbers of sequences with low affinities in Pse (692 - 310 = 382 for Bcd and 2001 - 685 = 1316 for Kr, Table 2) are too large to be explained by misclassification of high affinity sequences (*p*<10^−15^ for both factors assuming the misclassification rates at 0.13 for Bcd and 0.22 for Kr, Binomial test). Correcting for false positives and false negatives, we estimated the fraction of bound sequences with conserved affinities by multiplying the observed fraction with (1 – false positive rate), to account for false positives, and by dividing the result by sensitivity, to account for false negatives. This led to the estimates that 46% of Bcd targets and 55% of Kr targets remain bound by their respective factors in the Pse genome. Interestingly, even if we limit to sequences not only bound by TFs in the ChIP-chip experiments, but also adjacent to some gene transcribed in blastoderm, the fractions of sequences with conserved affinities are virtually unchanged (compare the last two rows in Table 2). These results suggest a high level of turnover of TF-binding across Mel and Pse genomes. While similar observations have been made before in other organisms [2], [58], what is striking here is that even the strongest bound sequences whose nearest genes are transcribed (a sign of regulatory functions) display low levels of affinity conservation across species.

**Figure 5:**
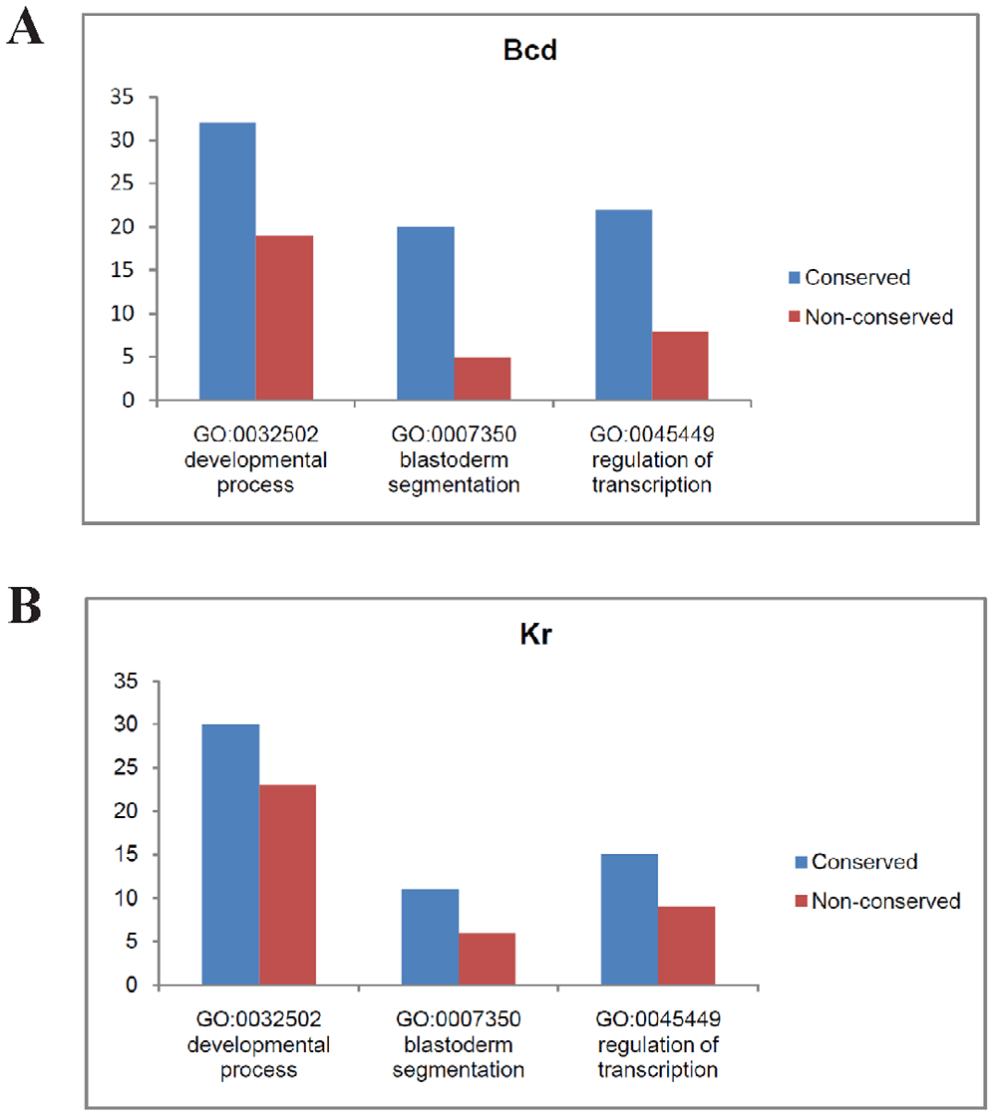
The functional characterization of sequences bound by Bcd and Kr (1% FDR) in the *D. melanogaster* genomes. The sequences are divided into the Conserved and Non-conserved groups, depending on whether the orthologous sequences in *D. pseudoobscura* also have high binding affinities. The GO annotations of the nearest genes of sequences in each group are analyzed (only top 50 sequences in each group, ranked by the strength of binding).

While there may be alternative interpretations of the lack of conservation (see Discussion), one simple hypothesis would be that TF binding, and even with the transcription of adjacent genes, is not sufficient to establish functionality. We reasoned that if this is true, we might be able to filter the non-functional sequences from all bound ones by testing the binding affinities of the orthologous sequences, an idea successfully applied in yeast studies [2]. We classified the bound sequences in Mel (at 1% FDR, with the extra requirement of being adjacent to some expressed gene) into two categories: those with high predicted affinities in Pse (Conserved group) and those with low affinities (Non-conserved group). We extracted the adjacent genes of these two groups of sequences to analyze the putative functions of these sequences (we limit to the top 50 sequences in each group as the total number of genes in each group is large). We found that the sequences in the conserved group are much more likely to be associated with genes in the relevant functional classes, such as “developmental processes” (Figure 5). These results suggest that by using the predicted affinities of orthologous sequences as a filter, one can enrich the functional sequences in the results from genome-wide binding experiments. This approach of improving function sequence prediction from conservation is different from the more common approach of using nucleotide-level conservation, which is sensitive to alignment between orthologous sequences.

## Discussion

In this work, we adapted the theoretical models pioneered by Shea-Ackers [29] and formulated by Buchler et al. [30] to the analysis of large-scale TF binding data. Different from these previous works, we explicitly expressed the expected number of TFs bound by a given regulatory sequence, and thus derived a variation of the Shea-Ackers model suitable for analysis of genome-wide binding data. We developed a dynamic programming algorithm that efficiently computes the binding affinity of any sequence. We provided software, STAP, to automatically learn the best models from the binding data. Through extensive evaluations, we demonstrated that this is an effective computational framework to extract information from and extrapolate over TF-DNA binding data.

STAP was applied to several important analysis tasks, including comparison of TF binding profiles, identification of TF interactions, studying the effect of binding site arrangement (regulatory grammar) and prediction of TF target sequences. These tasks are commonly encountered in analysis of genome-wide data, and we believe STAP offers key benefits over existing methods. First, STAP was applied to compare several putative Nanog motifs. Such functionality can be useful, for example, when one needs to compare outputs from multiple motif-finding programs or from different experiments. Furthermore, when multiple factors access the same target regions, STAP is able to disentangle the effects of confounding factors. This was demonstrated in the analysis of Nanog-bound sequences, which are often bound by Oct4 and Sox2 as well. Second, we took advantage of the new method to predict TF-TF interactions. Similar analyses were done previously by first predicting the binding sites of the pair of motifs, and then analyzing the co-occurrence pattern of two types of sites [27], [28]. Co-occurrence based analysis does not utilize the measured TF-binding intensities, sacrificing a significant amount of available information. Co-occurrence based analysis also requires the explicit annotation of binding sites, a task known for its inaccuracy. Weak binding sites were shown to contribute significantly to TF binding [23], [34], making a binary demarcation of sites and non-sites more problematic. Thirdly, STAP was applied to test different regulatory rules for binding site arrangement. This task has been gaining attention from the community [11], [12], but a computational tool for addressing this challenge has been missing so far. Finally, we demonstrated that STAP is able to make more accurate predictions of TF targets in new sequences than other state-of-the-art programs. This capability enables the study of the evolution of TF binding across species despite that the binding data are often available in only one species. We also found that limiting to sequences with conserved affinities would improve the identification of functional TF targets.

The recent work by Segal et al. [23] also uses the thermodynamic model to predict the functional properties (expression patterns) of DNA sequences, and it is worthwhile to point out the similarity and the difference between the two papers. Both Segal et al. and this work rely on the same thermodynamic framework of Buchler et al. [30] to model TF-DNA interactions as well as cooperative DNA binding by multiple TFs. In the algorithmic side, both use dynamic programming to optimize the computational task, which is also a familiar technique in statistical mechanics (known as the transfer matrix method), and has been used before for similar calculations involving *cis*-regulatory sequences [59], [60]. These similarities are not surprising as both attempt to capture the same underlying physics. There are two main differences. Segal et al. uses a logistic function as the expression “readout” of any molecular configuration (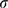 in our notation) and predicts the expression of the sequence as the average readout over all configurations. The downside of this approach is that the logistic function has no connection to thermodynamics, and the computation involves expensive sampling. In this work, the relevant quantity we compute has a clear physical interpretation: the average number of TF molecules bound to the sequence. This also enables the derivation of dynamic programming, which is far more efficient than sampling. The other main difference lies in the intended applications of the models. STAP was applied to questions that were not addressed previously, such as the characterization of rules of cooperative interactions and evolution of TF-target relationship.

Combinatorial gene regulation by definition involves the relationship among different transcription factors. However, how such relationships should be defined and inferred is not clear in practice. We believe it is important to distinguish among three types of relationship between a pair of transcription factors (Figure S8): (A) co-localization of two factors as revealed by ChIP experiments; (B) direct binding of two factors to the neighboring DNA sites (co-binding) and (C) cooperative interaction of two factors bound in the neighborhood. Note that these three classes correspond to progressively more specific relationships. Co-localization of two TFs in a ChIP experiment may be due to co-binding, or due to one of the TFs being bound to DNA and recruiting the other TF (without the latter directly binding to DNA). Similarly, when two factors bind to adjacent sites on DNA (co-binding), they may not actually interact with each other, i.e. no cooperative interactions. The different results we obtained from our co-localization analysis, from motif enrichment test using Clover and from our identification of cooperative factors may partly come from these distinctions (compare Tables S1, S5 and Table 1). This picture of a hierarchy in the relationships of TFs (in the context of DNA binding) suggests that it is important to interpret the results in a way that is appropriate for the type of analysis performed.

We assumed that cooperative interactions are due to protein-protein interactions, but this may not always be true. For example, the factor *B* may stimulate DNA-binding of the factor *A* through chromatin modification that makes DNA more accessible. This point has also been commented before [59]. It is difficult to distinguish different mechanisms of cooperative interactions when only DNA binding data is available. This is important for interpreting the results, as the predictions may not be confirmable through protein-protein interaction assays. In addition, this suggests that the cooperative interactions, as defined by stimulated effects of DNA binding on another factor, may not be symmetric. In the example we cited above, the factor A itself may not modify chromatin structure, thus has no effect on DNA binding affinity of the factor B.

We studied the effect of binding site orientation and relative distance on the cooperative TF interactions. Because the effect is likely to be subtle, we focused on the TF pairs with the strongest signals in the data. We did not found evidence supporting rigid rules, such as the periodicity of distance (in the range of period tested). This may suggest that the interactions may occur indirectly, rather than through physical protein-protein interactions, such as the well known case of lambda repressor [5]. If a TF modifies the chromatin structure through chemical modifications of histones or remodeling of nucleosomes, the effect of this TF on other TFs will be less specific (as it could affect all binding sites in the neighborhood) and less likely to follow strict rules. We recognize there are several limitations in our methodology: only several forms of cooperative functions were tested while the actual function may be much more complex; and in the thermodynamic model, only immediately adjacent binding sites may interact with each other, an assumption taken for the ease of computation without much theoretical justification. These limitations coupled with the fact that only five TF pairs were tested in a single dataset limit our ability to extrapolate any general regulatory rules. Still, the STAP method is relatively sensitive, as demonstrated by the large effect of *d*_max_ and the amplitude parameters we observed (Figure 4), and represents one concrete step towards an important but difficult problem.

STAP can be applied to learn TF binding models in one species and extrapolate to another species. This enabled the study of the evolution of sequences in terms of their interaction with TFs. That TF-binding of DNA sequences may not be constrained evolutionarily has been reported in yeasts and mammals [58], [61]. In *Drosophila*, it was reported that important TFBSs are subject to turnover across related species [31], [62]–[64]. The analysis based on the conservation of individual binding sites, however, does not address the question whether a promoter or enhancer, which typically have multiple binding sites, would have conserved functionality or not, as the gains and losses of binding sites in the neighborhood may compensate each other so that the overall affinity remains largely unchanged [56], [64], [65]. By predicting binding affinities directly in the Pse genome, without relying on sequence alignment and tracing the fate of individual sites, we showed that even the overall affinities are largely un-conserved. The fact that this also applies to sequences adjacent to transcribed genes adds another interesting dimension to the findings. One possible explanation is that these “biochemically active” sequences provide no evolutionary advantages, but merely serve as sequence “warehouse” for future functional elements [61]. Another possibility is that many of these sequences are functional, lineage-specific elements that evolve from adaptation to specific environment of *D. melanogaster* [66].

## Methods

### Biophysical Model of TF-DNA Interaction

Given a sequence *S*, our goal is to predict its binding intensity with the experimental TF, denoted as TF_exp_. We first scan the sequence with the position weight matrices of all relevant TFs (including TF_exp_ and possible cooperative factors) using very low thresholds to identify putative binding sites [4]. Thus our sequence would contain both strong and weak binding sites, instead of a single best match site for each factor. Note that this step is not absolute necessary as each position in theory can bind to any TF. We choose to discard those very weak sites only for the purpose of speeding up computation. For a binding site 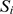, its affinity to its corresponding TF is given by [3]:
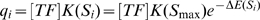
(1)where 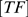 is the TF concentration, 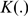 is the equilibrium constant of a site, 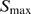 denotes the consensus sequence of this TF, and 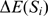 is the mismatch energy of 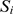 in the unit of 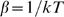. Note that 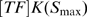 can be considered as a single TF-specific constant, denoted as 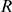 and the mismatch energy is related to the commonly used PWM matching score [3], [4]. Suppose *S* contains 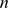 binding sites, a state 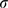 of *S* is represented by an 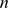-bit vector, where 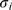 represents whether the 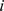-th site is occupied by its corresponding TF (equal to 1) or not (0). The probability of 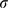, 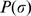, is determined by its Boltzmann weight, 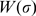 [30]:
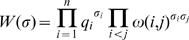
(2)where 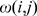 denotes the interaction between the two sites 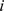 and 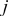 when both are occupied (Figure 1, the first column). The state where two overlapped binding sites are occupied simultaneously is not allowed, i.e. its weight is zero. Sites 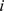 and 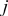 may denote the motifs of the same TF, and therefore self-cooperativity (the cooperative interactions among binding sites of the same TF) is accounted for in the model. Note that the interaction may depend on the arrangement of the binding sites. Our default model of interaction is a simple binary model: the bound factor at position 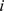, 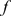, and the bound factor at position 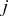, 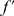, can interact with constant 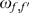 if the distance of their binding sites is less than 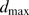. Basically, the above equation states that the weight of a particular state has two components: one from binding of TF to individual sites; and the other from cooperative interactions among bound TFs. In theory, any two bound TF molecules can form interactions; in reality, however, this is quite unlikely to be true. So we make the assumption that only two adjacent bound TF molecules can interact with each other. We assume that the binding affinity of the whole sequence to TF_exp_ (denoted as index *k*) is proportional to the expected number of bound molecules of 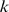, averaging over all states:
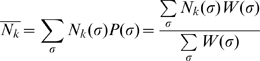
(3)where 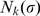 is the number of bound molecules of 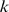 in 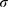 (Figure 1, the second column).

Because the number of states is exponential to the number of sites in a sequence, the brute-force computation of the above quantity is expensive. The computation of the partition function (the denominator) follows the transfer matrix method in statistical mechanics and is similar to the dynamic programming algorithms in other related work [23], [59], [60]. We show that dynamic programming can also be applied to compute the summation in the numerator, due to the simplicity of the functional form of 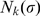 (note that summation of some function defined on σ may not always be solvable by dynamic programming). Let 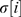 be one configuration up to the site 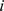, where 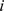 is bound by its cognate TF *f_i_*, we define: 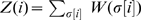 and 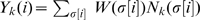. We have the following recurrence equations:
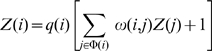
(4)

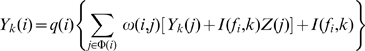
(5)where 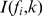 is the indicator variable of whether 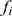 is equal to 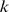. Finally we have: 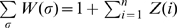 and 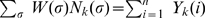. The details of deriving these equations can be found in Text S1.

### Implementation and Model Fitting

We implemented this model and the fitting procedure in the software, STAP. It can be used for analyzing both ChIP-chip and ChIP-seq data, available at: http://biocomp.bioen.uiuc.edu/STAP.

STAP takes as input a set of sequences and their measured binding intensities to TF_exp_, a set of TF motifs (including TF_exp_) and learns TF-binding models that can be used to predict binding affinity of any new sequence. A TF-binding model consists of two parts: the set of cooperative factors, and the free parameters, which include 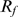 for each factor 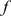, and the interaction parameters between the primary factor and any cooperative factors (including self-cooperative interactions). We note that when there are more than one cooperative factor, we do not allow interactions among these factors, as doing so will greatly increase the number of parameters (quadratic to the number of factors), and we may not be able to estimate them since we only have binding data for the primary factor.

At the first step of creating the binding model, we learn the motifs in the input motif collection that are cooperative to TF_exp_. For each of these motifs, we calculate the correlation coefficient of the model including this motif as well as TF_exp_. We estimate the statistical significance of this value by comparing with a null distribution constructed from randomized motifs. Specifically, we choose randomly a motif from a background motif library (we used JASPAR [36]), which could be different from the input motif collection, and then randomly shuffle the columns of this motif. The correlation coefficient of the model using this random motif and the primary factor will be estimated. The null distribution consists of the correlation values from 1000 randomized motifs. We use *p* value 0.05 as the threshold for significance judgment. After learning all significant motifs, we combine them into a single model and estimate the model parameters. For parameter estimation, we use the combination of the Nelder-Mead simplex method and the quasi-Newton method (the BFGS algorithm), both provided in the GNU Scientific Library [23], [67]. We alternate the two optimization methods until the solutions converge (as defined by the respective criterion of the two methods) or a specified number of alternations are reached. This approach is not guaranteed to find the global optimum, but we find through simulation that it usually produces reasonable solutions, while the global optimization method we tested, Simulated Annealing, is too slow for our purpose.

When running STAP on a TF dataset from ChIP-chip or ChIP-seq experiments, we generally need to use only a subset of data for training the binding model, while the rest can be used as testing data. In our experiments with both stem cell ChIP-seq data and *Drosophila* ChIP-chip data, we first identify the peak positions of the strongest bound regions (provided in both cases from our data sources) and extract the surrounding sequences, defined as 250 bp upstream/downstream of the peaks. Since these sequences only represent regions bound by TFs, we also add an equal number of sequences that do not show significant binding, chosen randomly from the genome. The binding affinities of these negative sequences are not always available, so we use some value below the lowest binding affinity among all bound sequences, as the substitute of measurements. In our experiments, the size of the training data is 1000 sequences (500 for both positive and negative sets). Our construction of testing data is similar: we choose the next 500 bound sequences and 500 random unbound sequences.

### Models of Cooperative Interactions

We denote the cooperative interaction between two bound factors, 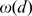, where 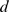 is the distance between two sites. It may also depend on the orientations of the two sites. Let 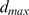 be the maximum distance where two bound factors can interact. We consider several forms of the function 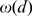. Under the Binary function, the interaction term is equal to a constant, 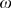 if 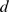 is less than 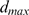; and 1.0 otherwise (no interaction, corresponding to free energy at 0). The orientation bias is modeled by multiplying a constant to 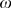 if the two sites are at different strands. The Linear function is defined by:
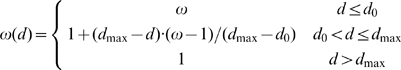
(6)


The orientation bias is modeled similarly. To derive the Periodic function, we assume that the free energy of interaction consists of a constant plus a term corresponding to the energetic cost of DNA looping. Following [52], the effective interaction between two factors *A* and *B* is given by:
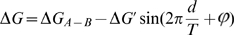
(7)where 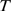 is the period, 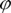 is the phase parameter and 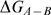 and 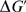 are constants. The interaction weight is 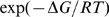 when 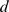 is less than 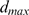 and 1.0 otherwise. The amplitude parameter we used is the amplitude of the interaction weight, which is also a periodic function. Also note that 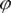 can in fact take two values, depending on whether the two sites are in the same orientation.

### Learning the Interaction Model between Two TFs

In studying the effect of binding site arrangement on TF interaction, we adopt a different model fitting procedure. Suppose we want to study the interaction of the factors A and B. We estimate a single set of parameters: 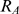, 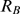 and the relevant interaction parameters (depends on how we model their interaction) from the binding data of both factors. The objective function is the average correlation coefficients between predictions and observations in the two sets of sequences. Also we vary the interaction parameters to observe their effects on the predictability of the model, as shown in the text, instead of estimating single optimal values. We note that such procedure is not applicable to fitting a “global” model of a large number of TFs (e.g. all 12 TFs in the mouse ESC dataset). In that case, the number of possible interactions is probably too large (66 in the ESC case) to be reliably estimated. Our software, however, does support estimating the global model when the number of factors is small (less than four, for instance).

### Data Used in *Drosophila* ChIP-Chip Analysis

We downloaded the processed ChIP-chip data of Li et al. [16], at both 1% FDR level and 25% FDR level, This dataset also includes the information of the nearest genes of the bound sequences and whether they are expressed in blastoderm. The random unbound sequences were extracted from the genome of *D. melanogaster*, Release 4 [16], and those overlapped with coding regions or bound regions at 25% FDR were removed. The known enhancers were taken from REDFly with the constraint that they must function in blastoderm development [68]. We extracted the orthologous sequences of all Mel sequences in the Pse genome using the alignment provided at UCSC [69]. The binding profiles of the factors Bcd and Kr were taken from the results of *in vitro* bacterial one hybrid (B1H) experiments [55]. When training the binding models, we used a collection of 66 motifs to learn the putative cooperative factors to Bcd and Kr. This collection is constructed by combining motifs from B1H experiments [55] and from DNA footprinting analysis [70].

## Supporting Information

Text S1 Additional details of experimental procedures and the algorithms.
(0.05 MB PDF)

Figure S1 ChIP-seq and ChIP-qPCR signals. Independent ChIP-qPCR experiments on randomly selected binding regions of Suz12 and Nanog generated highly correlated signals with the counts of overlapping ChIP-seq tags.
(0.08 MB PDF)

Figure S2 Motifs identified by MEME. For all factors except Oct4, Sox2, Nanog and E2f1, we ran MEME on the top 100 regions from ChIP-seq experiments (defined by 30 bp upstream and downstream of the peaks). For Oct4, Sox2 and Nanog, we ran MEME on all regions bound exclusively by Oct4, Sox2 and Nanog, respectively (i.e., for Oct4, we only consider regions bound by Oct4, but not Sox2 and Nanog; and similarly for Sox2 and Nanog). For E2f1, MEME failed to produce any specific motif, so we used the motif in the Transfac database.
(0.12 MB PDF)

Figure S3 Comparison of three versions of the Nanog motif: He09 - the one described in this paper, Mitsui03 from [40], Loh06 from [41]. The performance of a motif is assessed by the correlation coefficient of the model that uses this motif to fit the data of overlapping sequence counts of the 500 Nanog bound regions. We created the null distribution of the performance (the histogram) from 1000 random permutated motifs.
(0.06 MB PDF)

Figure S4 EMSA experiments of five genomic regions with high similarities to the new Nanog motif. Probes 1 to 5 correspond to the genomic regions 1 to 5 in Table S2. Probes P and N are positive and negative control probes, respectively. Negative control region: chr12:122668133–122668172 (mm8). Positive control region: chr18: 46513245–46513285 (mm8).
(0.08 MB PDF)

Figure S5 Mutation results do not depend on the wild-type binding sites. A subset of mutations chosen from Table S3 were repeated on two independent wild-type sequences. EMSA results of these mutated sequences are shown. The two independent wild-type sequences in the mutagenesis analysis generated similar results.
(0.11 MB PDF)

Figure S6 ROC curves comparing the performance of three methods for classification of TF target sequences in the ChIP-seq data. Red - STAP, purple - Clover + Cluster-Buster, blue - Clover + Stubb, black - Random classifier. For evaluation of Cluster-Buster and Stubb, the Clover program is run first on the training data to extract a set of overrepresented motifs, which will be used as inputs of Cluster-Buster and Stubb.
(0.64 MB PDF)

Figure S7 The effect of binding site arrangement on TF interactions. The left column shows the results under the Binary model of interaction: the relationship between model performances, measured by correlation between predictions and observations, and the distance parameter (maximum distance, measured in bp, where two factors can interact along DNA sequence). For each value of the distance parameter, two models are compared: one in which the orientation bias parameter is optimized, and the other not allowing the bias. The right column shows the results under the Periodic model of interaction: the relationship between model performances and the amplitude parameter (the change of the interaction strength within a period). Only two values of periodicity are shown.
(0.05 MB PDF)

Figure S8 Co-localization, co-binding and cooperative interactions between two TFs. (A) Co-localization without co-binding. The molecule of B is recruited to DNA by its interaction with a molecule of A that is already bound to the sequence. (B) Co-binding without cooperative interaction. The molecules of A and B bind independently to the DNA sequence. (C) Cooperative binding of the molecules of A and B. The arrow indicates the interaction between two molecules.
(0.03 MB PDF)

Table S1 Pearson _Χ_2 statistics of TF co-localization test using ChIP-seq data of multiple TFs. The larger the _Χ_2 value, the stronger evidence of co-localization (statistically significant if _Χ_2>6.63, or p<0.01).
(0.03 MB PDF)

Table S2 Five Nanog ChIP-seq positive regions containing the new Nanog sequence motif. All chromosome coordinates refer to UCSC mm8 mouse genome assembly.
(0.01 MB PDF)

Table S3 Binding affinities between Nanog and its mutated binding sequences. These biding affinities were derived from EMSA results of the point mutations of the new Nanog motif. A conserved motif TGATGGC/GC/T was identified in the screen. +++ strong binding, + weak binding, − no binding. All the results were reproduced by at least two independent assays. The DNA binding domain of Nanog and the complete Nanog protein produced the same binding affinities.
(0.04 MB PDF)

Table S4 Significant ESC cooperative factors. Each motif is evaluated by the model including this motif as well as the experimental factor (if the motif is the experimental factor itself, only homotypic cooperativity will be considered). The third column shows the p value estimated from the training data, and the last shows the correlation of the model in another testing data set.
(0.05 MB PDF)

Table S5 The overrepresented motifs identified by Clover. For each TF in the first row, the top 500 bound sequences are analyzed by Clover. The threshold of motif is set as 7.
(0.01 MB PDF)
