# Supplementary material for: A Biophysical Model for Analysis of Transcription Factor Interaction and Binding Site Arrangement from Genome-Wide Binding Data
Source: PLoS One. 2009 Dec 1;4(12):e8155. doi: 10.1371/journal.pone.0008155 (PMC2780727; doi:10.1371/journal.pone.0008155)
Supplement: Text S1 — Additional details of experimental procedures and the algorithms. (0.05 MB PDF) [file pone.0008155.s001.pdf]

SUPPLEMENTARY METHODS

Testing TF co-localization

We took the ChIP-seq data from [1] and followed their procedure to identify peaks that are bound by a TF. Our goal is to test if a factor, *A*, co-localizes with another factor, *B*. This translates to the hypothesis that *A* sites which are adjacent to some *B* sites (250 bp in our experiments) are enriched among all sites of *A*. We estimated the expectation of this number as well as the expected number of *A* sites that are not adjacent to some *B* sites, assuming the distribution of *B* sites follows a Poisson distribution whose rate is the genome-wide density of B peaks. These expected numbers are compared with the observed numbers of peaks via Pearson's χ^2 test.

Expression of Nanog protein

Recombinant proteins of the Nanog (GST tagged) were used for the gel shift assays. The full length Nanog protein was cloned into the pET42b (Novagen) vector. The proteins were expressed and purified with GSH-sepharose beads (Amersham). Eluents were dialyzed against a dialysis buffer (10 mM Tris–HCl, pH 7.4, 100 mM NaCl, 10 mM ZnCl2 and 10% glycerol) at 4°C for 6 h. Proteins were stored at -80°C.Concentrations of proteins were verified with the Biorad protein measurement assay.

Electrophoretic Mobility Shift Assay (EMSA)

DNA oligonucleotides (Proligo) labeled with biotin at the 5' end of the sense strands were annealed with the antisense strands in the annealing buffer (10 mM Tris-HCl , pH 8.0, 50 mM NaCl, 1 mM EDTA) and purified with agarose gel DNA extraction kit (Qiagen). DNA concentrations were determined by the NanoDrop ND-1000 spectrophotometer. The gel shift assays were performed using a LightShift Chemiluminescent EMSA kit (Pierce Biotechnologies). 100 ng of protein were added to a 5ul reaction mixture (final) containing 1 ug of poly(dI-dC) (Amersham), 1 ng of biotinlabeled oligonucleotide in the binding buffer (12 mM HEPES, pH 7.9, 12% glycerol, 60mM KCl, 0.25 mM EDTA, 1 mM DTT). Binding reaction mixtures were incubated for 20 min at room temperature. Binding reaction mixtures were resolved on pre-run 6%native polyacrylamide gels in 0.5X Tris-buffered EDTA. Gels were transferred to Biodyne B nylon membranes (Pierce Biotechnologies) using Western blot techniques and detected using chemiluminescence.

Algorithm of computing TF-binding affinity of DNA sequences

In the first step, we compute the denominator in Equation (3) of the main text: $Z = \sum_{\sigma} W(\sigma)$. This algorithm is similar to the ones used in several previous publications [2, 3]. Let $\sigma[i]$ be one configuration up to the site i, where i is bound by its cognate TF f_i. We could decompose the configuration $\sigma[i]$: supposing the nearest site to i that is occupied in this configuration is j ($j < i$, $j = 0$ if no site is occupied before i), we have:

$$W(\sigma[i]) = W(\sigma[j])\omega(i,j)q(i) \tag{1}$$

We use $Z(i)$ to denote the total statistical weight of all configurations up to i, where the site i is occupied, i.e., $Z(i) = \sum_{\sigma[i]} W(\sigma[i])$. Summing over all $\sigma[i]$ in the above equation and plugging in the expression of $Z(j)$ lead to the following recurrence:

$$Z(i) = q(i)\left[\sum_{j \in \Phi(i)} \omega(i,j) Z(j) + 1\right] \tag{2}$$

where $\Phi(i)$ is the set of sites before i that do not overlap with i. In order to compute Z, we note that the last bound site in any configuration could be $1, 2, \cdots, n$ or no bound site. So we have: $Z = 1 + \sum_{i=1}^{n} Z(i)$.

Next we compute the numerator $Y_k = \sum_{\sigma} W(\sigma) N_k(\sigma)$. We define the variable $Y_k(i) = \sum_{\sigma[i]} W(\sigma[i]) N_k(\sigma[i])$. For any specific configuration $\sigma[i]$, we have:

$$W(\sigma[i]) N_k(\sigma[i]) = \left[W(\sigma[j]) q(i) \omega(i,j)\right]\left[N_k(\sigma[j]) + I(f_i, k)\right] \tag{3}$$

where $I(f_i, k)$ is the indicator variable of whether f_i is equal to k. Summing over all $\sigma[i]$ and plugging in the expressions of $Z(j)$ and $Y_k(j)$, we have the following recurrence:

$$Y_k(i) = q(i)\left\{\sum_{j \in \Phi(i)} \omega(i,j)\left[Y_k(j) + I(f_i,k) Z(j)\right] + I(f_i,k)\right\} \tag{4}$$

The last bound site could be $1, 2, \cdots, n$ (if no site is bound, no contribution to Y_k), so we have: $Y_k = \sum_{i=1}^{n} Y_k(i)$.

REFERENCES:

1. Chen, X., V.B. Vega, and H.H. Ng, *Transcriptional Regulatory Networks in Embryonic Stem Cells.* Cold Spring Harb Symp Quant Biol, 2008.
2. Segal, E., et al., *Predicting expression patterns from regulatory sequence in Drosophila segmentation.* Nature, 2008. **451**(7178): p. 535-40.
3. Hermsen, R., S. Tans, and P.R. ten Wolde, *Transcriptional regulation by competing transcription factor modules*. PLoS Comput Biol, 2006. **2**(12): p. e164.
