# Supplementary figures and images for: A Biophysical Model for Analysis of Transcription Factor Interaction and Binding Site Arrangement from Genome-Wide Binding Data
Source: PLoS One. 2009 Dec 1;4(12):e8155. doi: 10.1371/journal.pone.0008155 (PMC2780727; doi:10.1371/journal.pone.0008155)

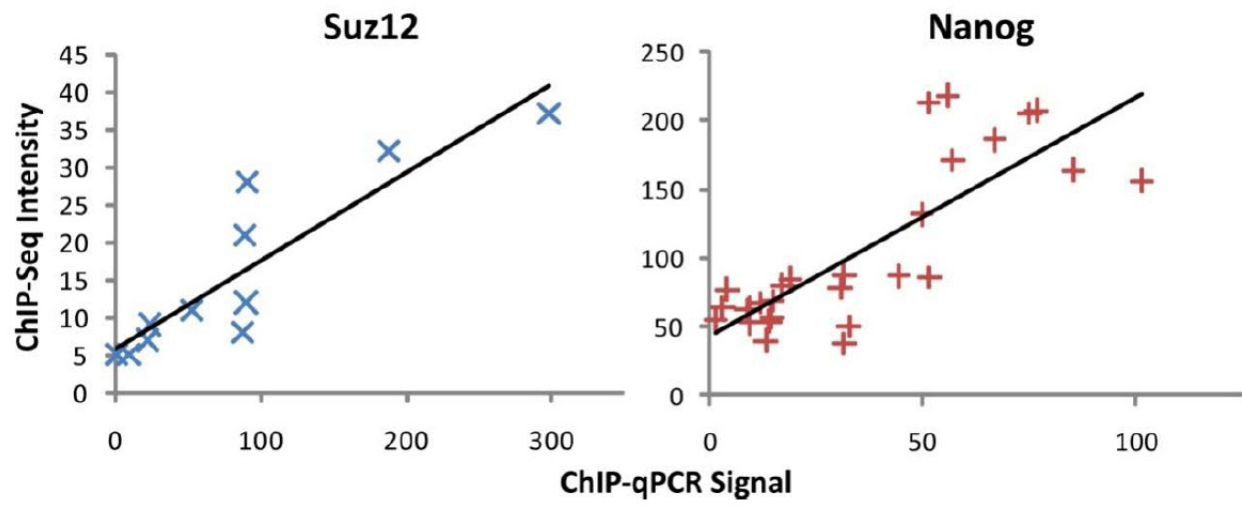

Supplement: Figure S1 — ChIP-seq and ChIP-qPCR signals. Independent ChIP-qPCR experiments on randomly selected binding regions of Suz12 and Nanog generated highly correlated signals with the counts of overlapping ChIP-seq tags. (0.08 MB PDF) [file pone.0008155.s002.pdf]

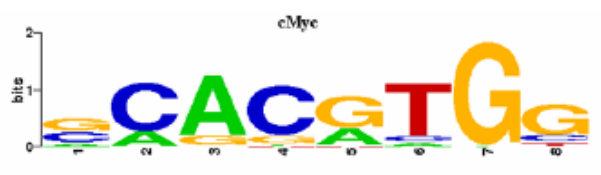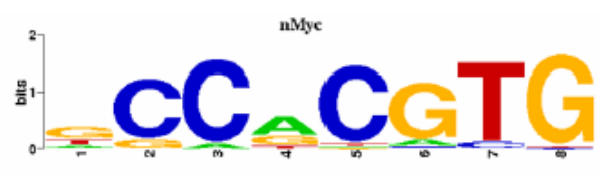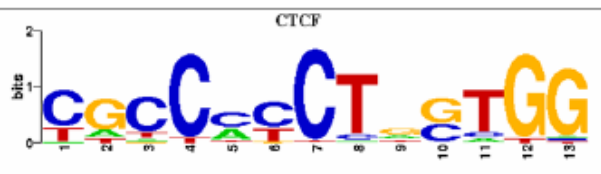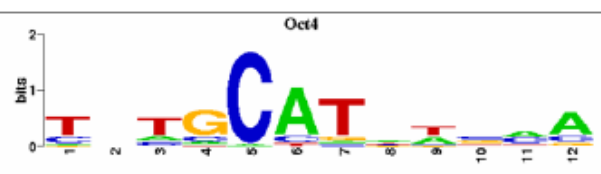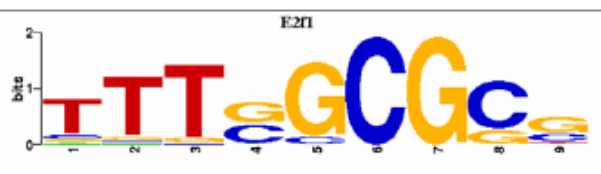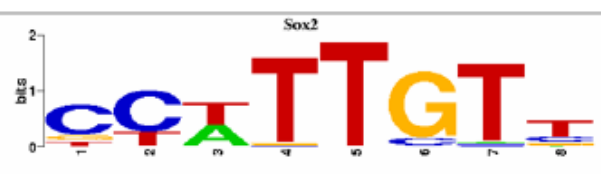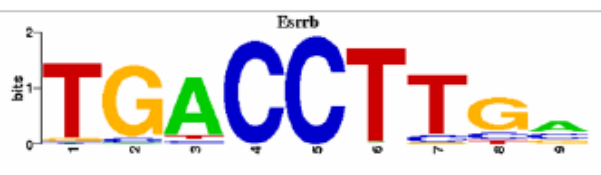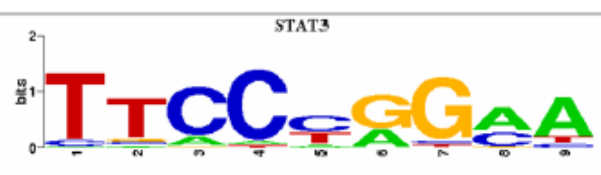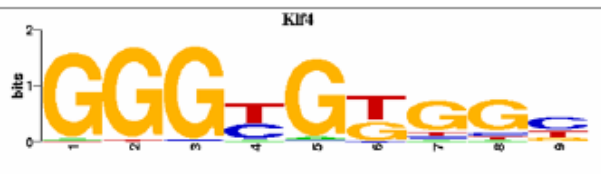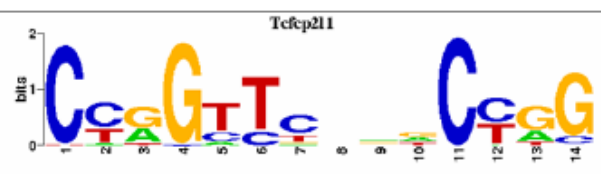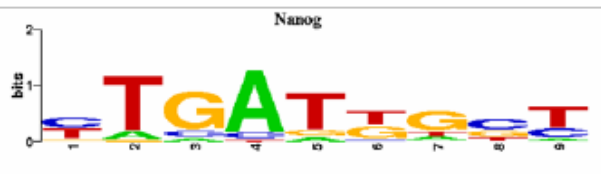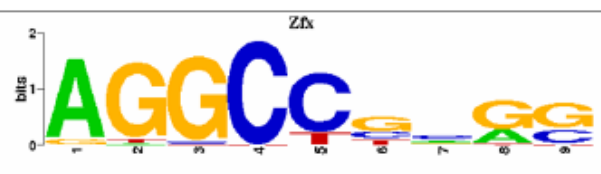

Supplement: Figure S2 — Motifs identified by MEME. For all factors except Oct4, Sox2, Nanog and E2f1, we ran MEME on the top 100 regions from ChIP-seq experiments (defined by 30 bp upstream and downstream of the peaks). For Oct4, Sox2 and Nanog, we ran MEME on all regions bound exclusively by Oct4, Sox2 and Nanog, respectively (i.e., for Oct4, we only consider regions bound by Oct4, but not Sox2 and Nanog; and similarly for Sox2 and Nanog). For E2f1, MEME failed to produce any specific motif, so we used the motif in the Transfac database. (0.12 MB PDF) [file pone.0008155.s003.pdf]

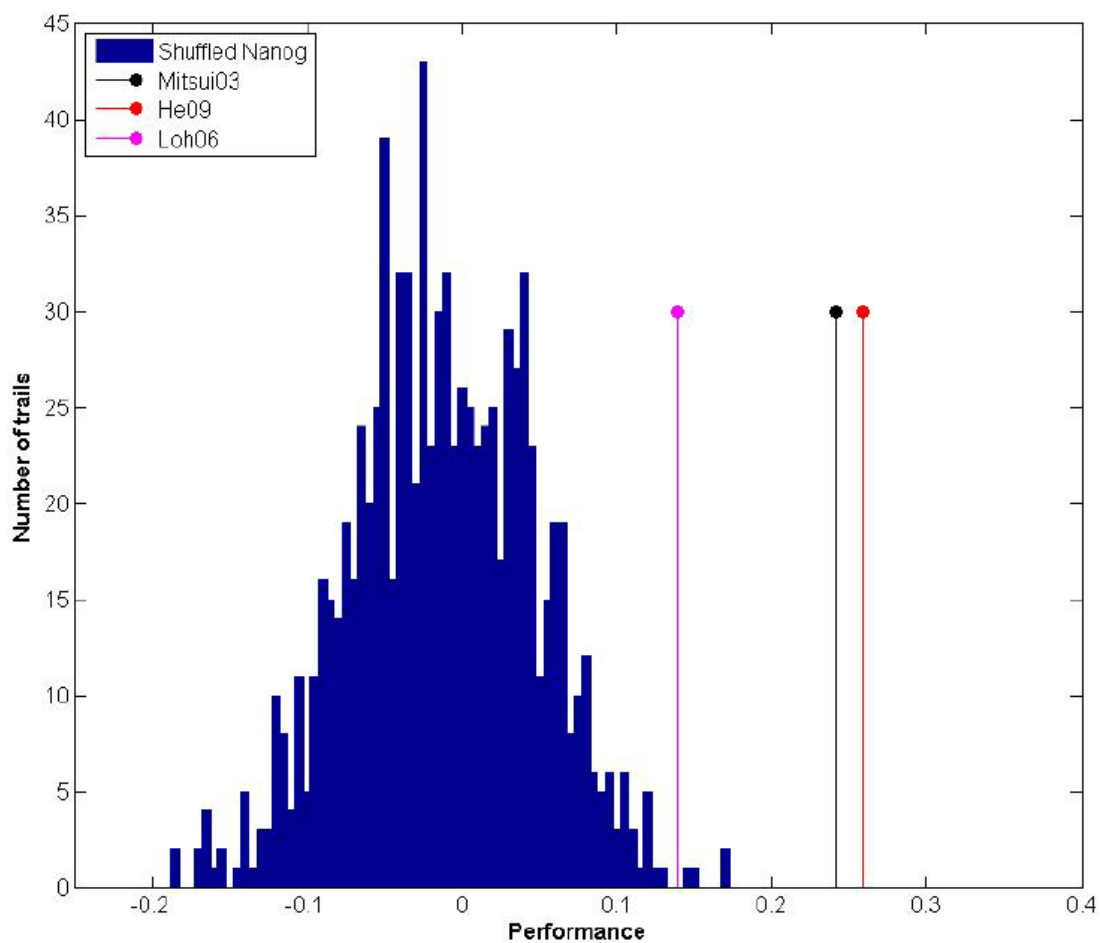

Supplement: Figure S3 — Comparison of three versions of the Nanog motif: He09 - the one described in this paper, Mitsui03 from [40], Loh06 from [41]. The performance of a motif is assessed by the correlation coefficient of the model that uses this motif to fit the data of overlapping sequence counts of the 500 Nanog bound regions. We created the null distribution of the performance (the histogram) from 1000 random permutated motifs. (0.06 MB PDF) [file pone.0008155.s004.pdf]

Nanog protein - + + + + + + +

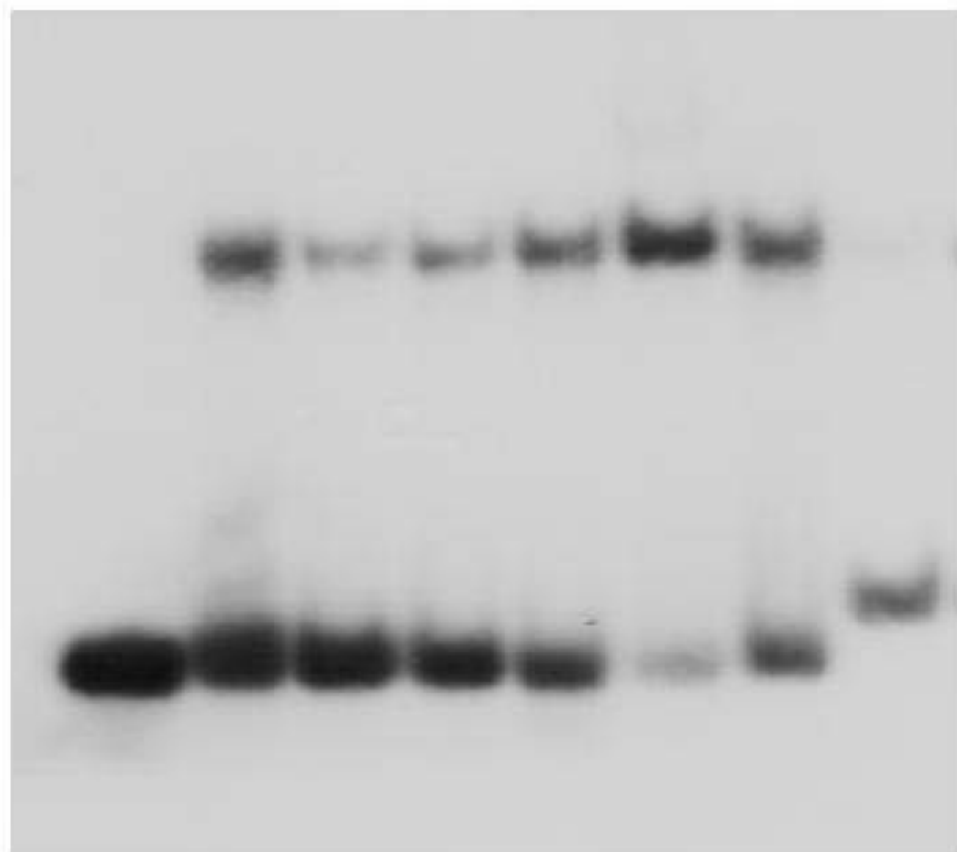

Probes 1 2 3 4 5 P N

Supplement: Figure S4 — EMSA experiments of five genomic regions with high similarities to the new Nanog motif. Probes 1 to 5 correspond to the genomic regions 1 to 5 in Table S2. Probes P and N are positive and negative control probes, respectively. Negative control region: chr12:122668133–122668172 (mm8). Positive control region: chr18: 46513245–46513285 (mm8). (0.08 MB PDF) [file pone.0008155.s005.pdf]

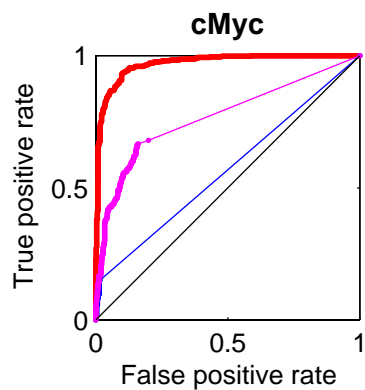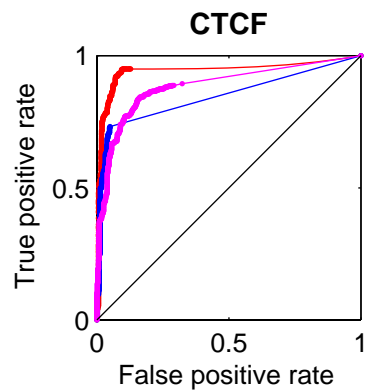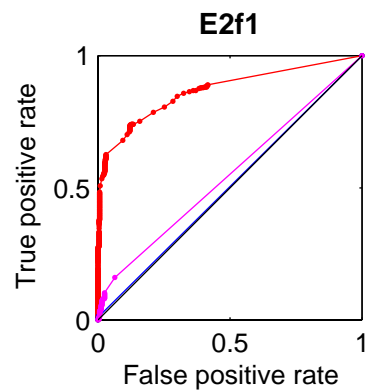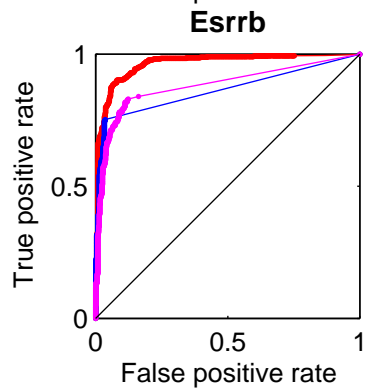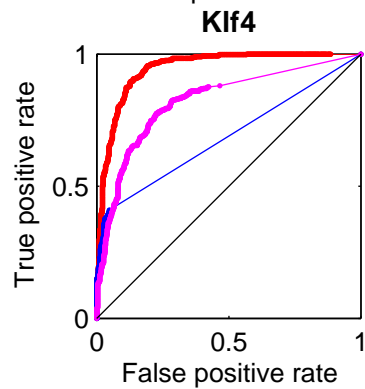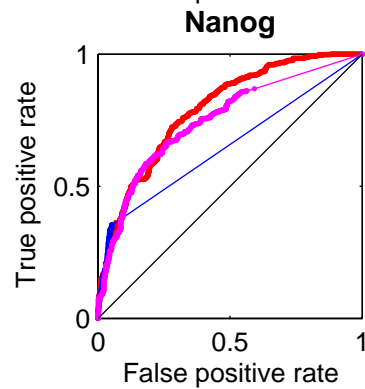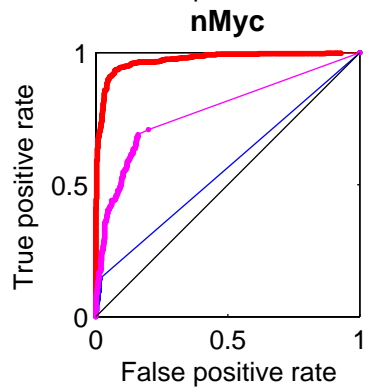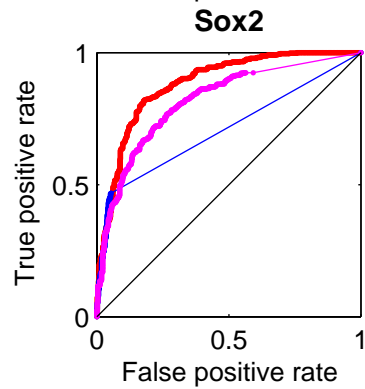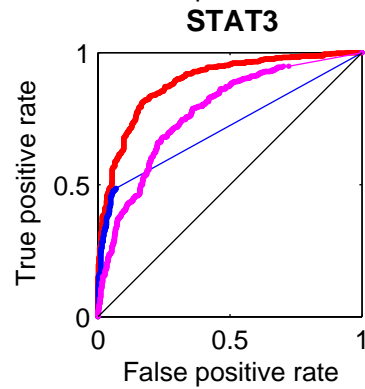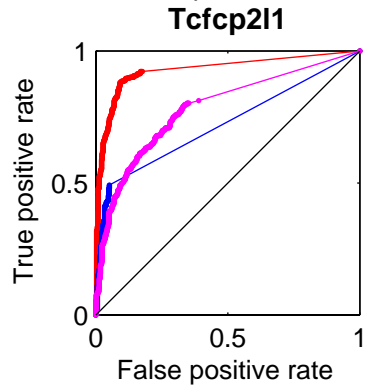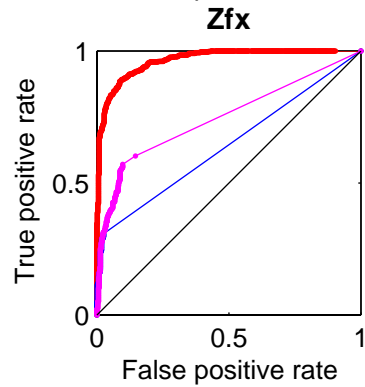

Supplement: Figure S6 — ROC curves comparing the performance of three methods for classification of TF target sequences in the ChIP-seq data. Red - STAP, purple - Clover + Cluster-Buster, blue - Clover + Stubb, black - Random classifier. For evaluation of Cluster-Buster and Stubb, the Clover program is run first on the training data to extract a set of overrepresented motifs, which will be used as inputs of Cluster-Buster and Stubb. (0.64 MB PDF) [file pone.0008155.s007.pdf]

**cMyc-E2f1**

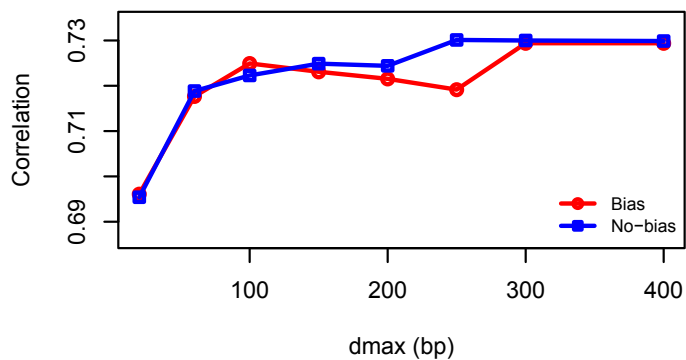

**cMyc-E2f1**

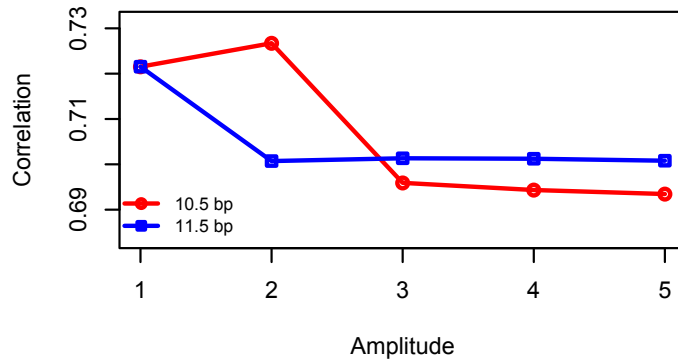

**STAT3-Klf4**

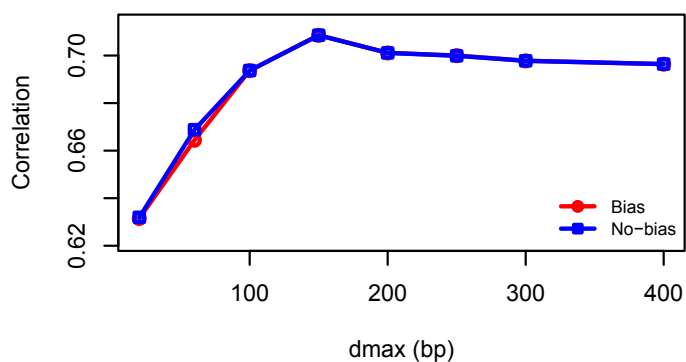

**STAT3-Klf4**

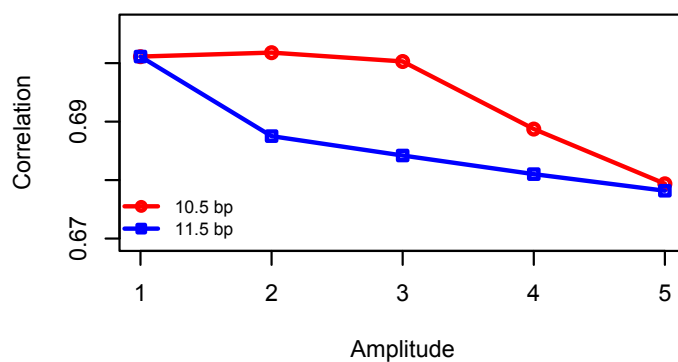

**Oct4-Zfx**

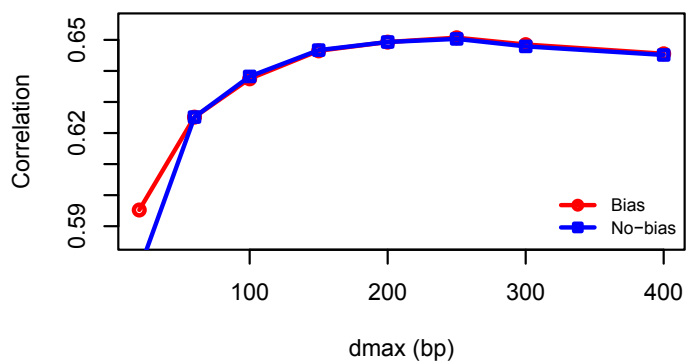

**Oct4-Zfx**

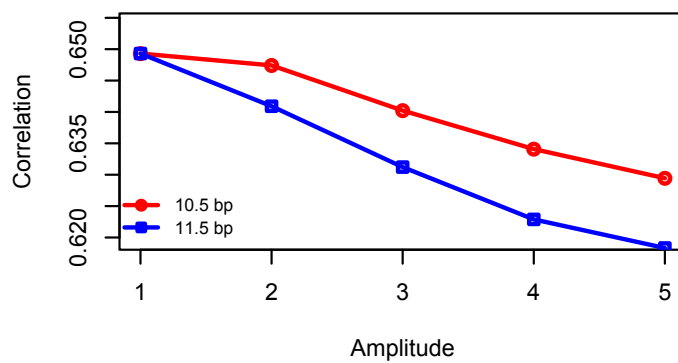

Supplement: Figure S7 — The effect of binding site arrangement on TF interactions. The left column shows the results under the Binary model of interaction: the relationship between model performances, measured by correlation between predictions and observations, and the distance parameter (maximum distance, measured in bp, where two factors can interact along DNA sequence). For each value of the distance parameter, two models are compared: one in which the orientation bias parameter is optimized, and the other not allowing the bias. The right column shows the results under the Periodic model of interaction: the relationship between model performances and the amplitude parameter (the change of the interaction strength within a period). Only two values of periodicity are shown. (0.05 MB PDF) [file pone.0008155.s008.pdf]

A

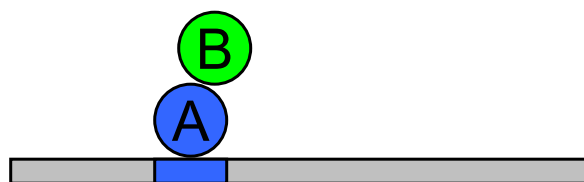

B

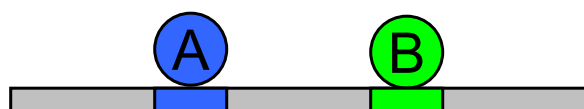

C

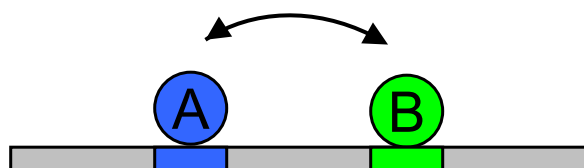

Supplement: Figure S8 — Co-localization, co-binding and cooperative interactions between two TFs. (A) Co-localization without co-binding. The molecule of B is recruited to DNA by its interaction with a molecule of A that is already bound to the sequence. (B) Co-binding without cooperative interaction. The molecules of A and B bind independently to the DNA sequence. (C) Cooperative binding of the molecules of A and B. The arrow indicates the interaction between two molecules. (0.03 MB PDF) [file pone.0008155.s009.pdf]
