# Supplementary material for: A Biophysical Model for Analysis of Transcription Factor Interaction and Binding Site Arrangement from Genome-Wide Binding Data
Source: PLoS One. 2009 Dec 1;4(12):e8155. doi: 10.1371/journal.pone.0008155 (PMC2780727; doi:10.1371/journal.pone.0008155)
Supplement: Figure S5 — Mutation results do not depend on the wild-type binding sites. A subset of mutations chosen from Table S3 were repeated on two independent wild-type sequences. EMSA results of these mutated sequences are shown. The two independent wild-type sequences in the mutagenesis analysis generated similar results. (0.11 MB PDF) [file pone.0008155.s006.pdf]

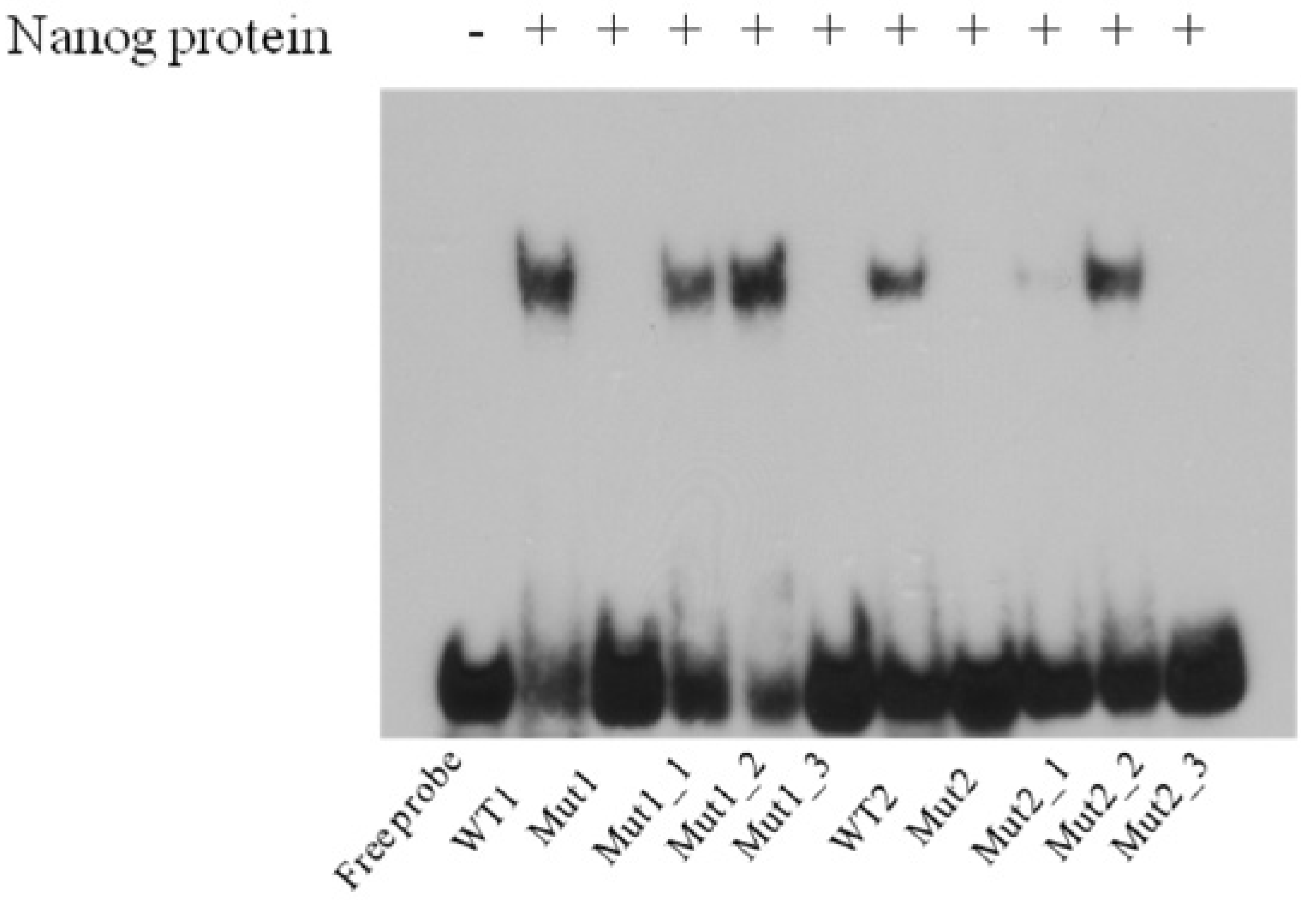


```
CAACCAGCCCTTGATGGCCCTCCTTGATGG    WT1
CAACCAGCCCTcacTGGCCCTCCTTGATGG    Mut1
CAACCAGCCCTcGATGGCCCTCCTTGATGG    Mut1_1
CAACCAGCCCTTaATGGCCCTCCTTGATGG    Mut1_2
CAACCAGCCCTTGcTGGCCCTCCTTGATGG    Mut1_3
CCTTTCAGCTCTGATGGGTTTCTTTCAGCT    WT2
CCTTTCAGCTCcacTGGGTTTCTTTCAGCT    Mut2
CCTTTCAGCTCcGATGGGTTTCTTTCAGCT    Mut2_1
CCTTTCAGCTCTaATGGGTTTCTTTCAGCT    Mut2_2
CCTTTCAGCTCTGcTGGGTTTCTTTCAGCT    Mut2_3
```
