# Supplementary material for: A Biophysical Model for Analysis of Transcription Factor Interaction and Binding Site Arrangement from Genome-Wide Binding Data
Source: PLoS One. 2009 Dec 1;4(12):e8155. doi: 10.1371/journal.pone.0008155 (PMC2780727; doi:10.1371/journal.pone.0008155)
Supplement: Table S1 — Pearson Χ2 statistics of TF co-localization test using ChIP-seq data of multiple TFs. The larger the Χ2 value, the stronger evidence of co-localization (statistically significant if Χ2>6.63, or p<0.01). (0.03 MB PDF) [file pone.0008155.s010.pdf]

	cMyc	CTCF	E2f1	Esrrb	Klf4	Nanog	nMyc	Oct4
cMyc	NA	3052.1	335600.7	14094.6	86628.3	2963.4	1319032	33918.6
CTCF	3019.2	NA	8794.4	6802.2	11134.8	183.8	12713.3	2448.5
E2f1	333960.1	8845.7	NA	60402.4	310860.1	32594.8	581847.8	79543.7
Esrrb	14020.7	6840.4	60385.8	NA	117170.5	76710.4	33659.8	43225.2
Klf4	86445	11230.7	311730.6	117532.2	NA	91453.1	208216.9	96856.7
Nanog	2957.4	186	32692.5	76959.5	91466.9	NA	8232.2	330694.9
nMyc	1317630	12835.4	584083.8	33800.3	208435.4	8239.8	NA	62577.3
Oct4	33915.4	2475.2	79928.6	43446.9	97052.7	331312.3	62638.2	NA
p300	924.7	13.1	4074.3	13590.3	21858.7	53793.2	1566.9	75788.3
Smad1	567	5.3	12614.1	46664.6	73722.1	320960.2	3175.9	296709.8
Sox2	3576.2	921.1	36522.2	57690.2	84894.6	797179.6	9262.4	642836.9
STAT3	11510.1	957.2	52990.6	50340.3	93933	80530.9	28791.1	86259
Suz12	75.2	138.7	210.2	2728	1290.2	0	1630	302.1
Tcfcp2l1	20204.7	9579.9	104882.1	105676.2	106140.7	79050.4	48377.3	51572.9
Zfx	206837	4882.1	386233.9	39353.9	113942.1	4272.2	326344.4	22499.2

	p300	Smad1	Sox2	STAT3	Suz12	Tcfcp2l1	Zfx
cMyc	923.9	566.6	3577.3	11507.2	75.2	20341.8	207243
CTCF	12.9	5.2	910.9	946.3	136.9	9542.3	4838.5
E2f1	4050.8	12543.7	36353.3	52717.1	209.1	105070.1	385097.6
Esrrb	13508.7	46393.1	57408.5	50066.8	2713.9	105836.8	39225.7
Klf4	21794.6	73518.6	84741.4	93711.2	1287.7	106629.8	113924.7
Nanog	53643.7	320124.2	795867.9	80352.8	0	79427.5	4272.2
nMyc	1564	3170.4	9255.3	28753.1	1628.6	48652.6	326636.7
Oct4	75719	296488.7	642976.1	86229.3	302.1	51915.6	22541.5
p300	NA	204955.9	88377.6	55222.1	6	8463.5	663.6
Smad1	204921	NA	408869.5	123817	5	32999.9	1135
Sox2	88277.6	408476.6	NA	87188	19.7	77136.6	5568.7
STAT3	55190.5	123767.3	87236.9	NA	4.4	44884.2	13264.6
Suz12	6	5	19.7	4.4	NA	2133.9	1667.3
Tcfcp2l1	8399.9	32758.3	76644.9	44572.9	2119.3	NA	39235
Zfx	661.7	1132	5559.3	13235	1664.3	39423.6	NA
