# Supplementary material for: A Biophysical Model for Analysis of Transcription Factor Interaction and Binding Site Arrangement from Genome-Wide Binding Data
Source: PLoS One. 2009 Dec 1;4(12):e8155. doi: 10.1371/journal.pone.0008155 (PMC2780727; doi:10.1371/journal.pone.0008155)
Supplement: Table S2 — Five Nanog ChIP-seq positive regions containing the new Nanog sequence motif. All chromosome coordinates refer to UCSC mm8 mouse genome assembly. (0.01 MB PDF) [file pone.0008155.s011.pdf]

Probe	Coordinate	strand	Sequence
**1**	chr13_3712191_3712231	+	TCCTGCAACCAGCCCTTGATGGCCCTCCTTGATGGCCCGC
**2**	chr19_21852503_21852543	+	GGATTCCTTTCAGCTCTGATGGGTTTCTTTCAGCTATTGA
**3**	chr4_41045395_41045435	-	AAGGCTTAGTCCTTGATGGGTTCTTTGTCATCCCAATCAA
**4**	chr6_112851211_112851251	+	TCACTTAATTCCTCCTTGATTGCTTTTCAAAAGCAATGTA
**5**	chr5_142668044_142668084	-	TGTGATTTATCCCTGATGGCCCATTAGTCCGGATGGTTTG
