# Supplementary material for: A Biophysical Model for Analysis of Transcription Factor Interaction and Binding Site Arrangement from Genome-Wide Binding Data
Source: PLoS One. 2009 Dec 1;4(12):e8155. doi: 10.1371/journal.pone.0008155 (PMC2780727; doi:10.1371/journal.pone.0008155)
Supplement: Table S3 — Binding affinities between Nanog and its mutated binding sequences. These biding affinities were derived from EMSA results of the point mutations of the new Nanog motif. A conserved motif TGATGGC/GC/T was identified in the screen. +++ strong binding, + weak binding, − no binding. All the results were reproduced by at least two independent assays. The DNA binding domain of Nanog and the complete Nanog protein produced the same binding affinities. (0.04 MB PDF) [file pone.0008155.s012.pdf]

ID	Sequence	Mutated position	Binding affinity
1	CTGATGGCC (WT)		+++
2	CcacTGGCC	2-4	-
3	CcGATGGCC	2	+
4	CaGATGGGT	2	+
5	CgGATGGGT	2	+
6	CTaATGGCC	3	+++
7	CTtATGGCC	3	+
8	CTcATGGCC	3	-
9	CTGcTGGCC	4	-
