# Supplementary material for: A Biophysical Model for Analysis of Transcription Factor Interaction and Binding Site Arrangement from Genome-Wide Binding Data
Source: PLoS One. 2009 Dec 1;4(12):e8155. doi: 10.1371/journal.pone.0008155 (PMC2780727; doi:10.1371/journal.pone.0008155)
Supplement: Table S4 — Significant ESC cooperative factors. Each motif is evaluated by the model including this motif as well as the experimental factor (if the motif is the experimental factor itself, only homotypic cooperativity will be considered). The third column shows the p value estimated from the training data, and the last shows the correlation of the model in another testing data set. (0.05 MB PDF) [file pone.0008155.s013.pdf]

TF	coFactor	P-value	Test corr.
**cMyc**	cMyc	NA	0.59
	E2f1	0.004	0.82
	Klf4	0.040	0.76
	Zfx	0.033	0.78
**CTCF**	CTCF	NA	0.82
**E2f1**	E2f1	NA	0.63
	Nanog	0.048	0.65
**Esrrb**	Esrrb	NA	0.76
	Zfx	0.003	0.78
**Klf4**	Klf4	NA	0.76
	CTCF	0.000	0.74
**Nanog**	Nanog	NA	0.23
	Klf4	0.012	0.38
	Sox2	0.000	0.39
	Zfx	0.050	0.35
**nMyc**	nMyc	NA	0.73
	E2f1	0.005	0.83
**Oct4**	Oct4	NA	0.42
	E2f1	0.029	0.48
	Klf4	0.032	0.52
	Zfx	0.017	0.52
**Sox2**	Sox2	NA	0.49
	Klf4	0.014	0.59
	Oct4	0.039	0.54
	Zfx	0.045	0.57
**STAT3**	STAT3	0.810	0.48
	E2f1	0.049	0.56
	Klf4	0.004	0.63
	Zfx	0.039	0.58
**Tcfcp2l1**	Tcfcp2l1	NA	0.75
	Erssb	0.011	0.76
**Zfx**	Zfx	NA	0.80
