# Supplementary material for: A Biophysical Model for Analysis of Transcription Factor Interaction and Binding Site Arrangement from Genome-Wide Binding Data
Source: PLoS One. 2009 Dec 1;4(12):e8155. doi: 10.1371/journal.pone.0008155 (PMC2780727; doi:10.1371/journal.pone.0008155)
Supplement: Table S5 — The overrepresented motifs identified by Clover. For each TF in the first row, the top 500 bound sequences are analyzed by Clover. The threshold of motif is set as 7. (0.01 MB PDF) [file pone.0008155.s014.pdf]

cMyc	cMyc, nMyc
CTCF	CTCF, Nanog
E2f1	N/A
Esrrb	Esrrb
Klf4	Klf4, Sox2, Esrrb
Nanog	Nanog, Sox2, Oct4, Esrrb
nMyc	cMyc, nMyc
Oct4	Oct4, Sox2, Nanog, Esrrb
Sox2	Sox2, Oct4, Nanog, Esrrb
STAT3	STAT3, Klf4, Sox2, Nanog, Oct4, Esrrb
Tcfcp2l1	Tcfcp2l1, Sox2, Esrrb
Zfx	Zfx
